# Aortic hemodynamic and morphological analysis before and after repair of thoracoabdominal aortic aneurysm using a G-Branch endograft

**DOI:** 10.3389/fphys.2023.1234989

**Published:** 2023-08-04

**Authors:** Jiabin Wang, Tingting Fan, Hongpeng Zhang, Yangyang Ge, Weihang Lu, Feng Liu, Dan Rong, Wei Guo

**Affiliations:** ^1^ The First Medical Centre, Department of Vascular and Endovascular Surgery, Chinese PLA General Hospital, Beijing, China; ^2^ Medical School of Chinese PLA, Beijing, China; ^3^ School of Biomedical Engineering, Capital Medical University, Beijing, China

**Keywords:** multibranched stent graft, thoracoabdominal aortic aneurysm, hemodynamics, morphology, thoracic endovascular aortic repair

## Abstract

**Background and objective:** The G-Branch endograft is a novel multibranched “off-the-shelf” device used to repair thoracoabdominal aortic aneurysms (TAAAs). This report describes the hemodynamic and morphological performance of the G-Branch endograft in a human patient with TAAA.

**Materials and methods:** We retrospectively reviewed the computed tomography angiography scans and clinical data of a woman in whom TAAA was treated using a G-Branch endograft. Patient-specific three-dimensional models were reconstructed, and computational fluid dynamics and morphological and hemodynamic indicators were analyzed before and after implantation of the device.

**Results:** From a morphological perspective, there was an increase in cross-sectional area in the G-Branch endograft and all bridging stent grafts over time. Blood flow was redistributed among the renovisceral arteries, with a decrease in flow rate in the celiac artery and an increase in the left renal artery. Laminar blood flow was smoother and more rapid after implantation of the G-Branch device and remained stable during follow-up. In the bridging stent grafts, flow recirculation zones were found in the bridging zones of the celiac artery and superior mesenteric artery as well as the distal sealing zones of both renal arteries. Furthermore, higher time-averaged wall shear stress and a lower oscillatory index and relative resident time were found in the G-Branch endograft and bridging stent grafts. Quantitative analysis showed obvious reduction in the surface area ratio of the elevated time-averaged wall shear stress area and surface area ratio of the relative resident time after G-branch implantation.

**Conclusion:** The revascularization of branch vessels occurred following G-branch implantation, with improvements arising not only from morphological changes but also from hemodynamic alterations. The long-term performance of the G-Branch endograft needs further investigation and clinical validation.

## 1 Introduction

Thoracoabdominal aortic aneurysms (TAAAs) are at high risk of rupture, and timely surgery is essential. TAAAs repair requires reconstruction of the renovisceral arteries, which can be performed via an open, hybrid, or endovascular approach. Minimally invasive total endovascular aortic repair has become the preferred treatment for TAAAs. “Off-the-shelf” multibranched endografts, one of the kinds of branched endografts, are now increasingly used for total endovascular aortic repair of symptomatic or ruptured TAAAs. The G-Branch endograft is one of four multibranched endografts (Oderich et al., 2019; Bilman et al., 2020; Kölbel et al., 2021; Ge et al., 2022). Unlike the other three endografts, the G-Branch includes two parallel inner branches for reconstruct the celiac artery (CA) and superior mesenteric artery (SMA). This conformation makes reconstruction of the CA and SMA relatively straightforward in a narrow space (Katsargyris et al., 2018).

Computational fluid dynamics (CFD) is a useful non-invasive method for assessment of aortic hemodynamics and provides insights into spatial-temporal variations in flow (Northrup et al., 2022). CFD has been fully used to evaluate hemodynamic performance of branched endografts repairing either aortic arch pathology or TAAAs. With regard to aortic arch pathology, many studies have demonstrated that aortic flow patterns were significantly altered by the branched endografts which caused increased spatial variation of wall shear stress in the ascending aorta and the arch (Zhu et al., 2019; Qiao et al., 2020; Li et al., 2023). But, the branches away from regions covered by the stent grafts seems have little effect on flow upstream or downstream (Zhu et al., 2019; Li et al., 2023). This phenomenon indicated that the branches seems just altered flow patterns in the regions covered by the branched endografts. When it comes to Branched endografts’ applications on TAAAs, different discoveries occurred. In a study by Kandail et al. (2015), implantation of branched stent grafts increased the complexity of flow in comparison with that in fenestrated stent grafts and led to larger flow recirculation zones (FRZs) in bilateral renal arteries, which were prone to thrombosis. Moreover, a study of the hemodynamic performance of physician-assembled endografts used to repair TAAAs by Liu et al. (2021) found a relationship between several hemodynamic parameters and in-stent thrombosis. Changes in morphology may also cause hemodynamic changes and *vice versa* (Parker et al., 2019). Tricarico et al. (2017) have shown that cross-sectional area (CSA) may predict stent graft occlusion.

To a large extent, renovisceral arteries reconstruction and their long-term patency have a huge impact on technical and clinical success of an “off-the-shelf” multibranched endograft in the repair of TAAAs. However, whether the conformation of the G-Branch can maintain a stable morphology and improve the hemodynamic environment remains unclear. Computational fluid dynamics research usually focuses on a single time point, so it cannot provide information on the temporal relationship between hemodynamics and vessel remodeling. Therefore, in this study, we investigated the hemodynamic and morphological performance of the G-Branch by patient-specific analysis.

## 2 Methods and materials

### 2.1 Image acquisition

**FIGURE 1:**
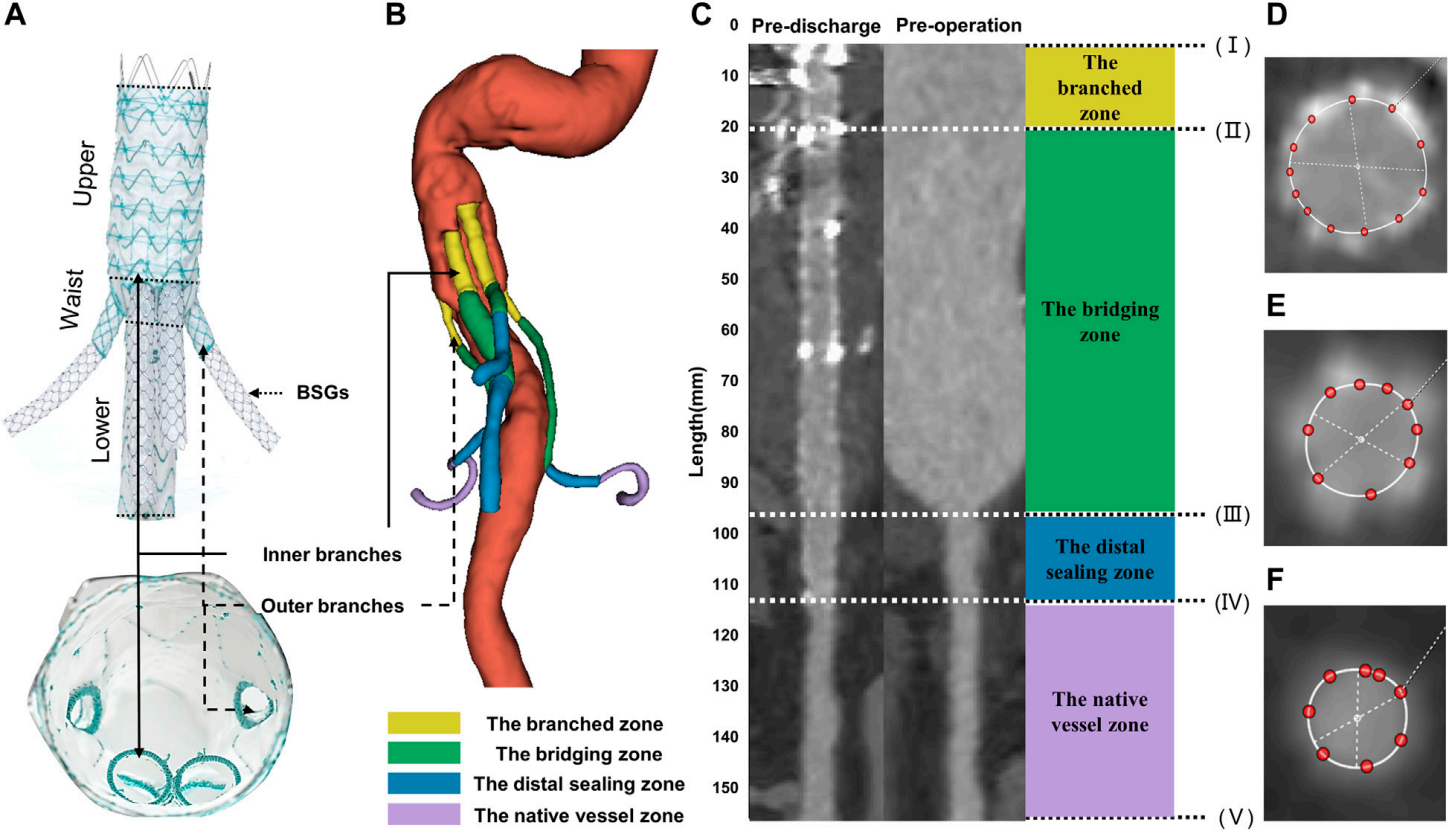
Illustrations of G-branch endografts and four zones in BSGs. **(A)** Compositions of G-branch endograft. **(B)** Four zones were plotted in a 3D model. **(C)** Vessel straightening and definition of four zones. Four zones were divided by five points, (I) the initial site of the BSGs; (II) the branched point; (III) the vessel branch point; (IV) the distal end of BSGs; (V) the bifurcation point. The branched zone between the initial sites of the BSGs (I) and the branched point (II); the bridging zone between the branched point (II) and the vessel branch point (III); the distal sealing zone between the vessel branch point (III) and the distal end of the BSGs (IV); and the native vessel zone between the distal end of the BSGs (IV) and the bifurcation point (V). **(D–F)** Cross sectional area measurements in BSGs and in renovisceral arteries. BSGs, bridging stent grafts.

The G-Branch (Lifetech Scientific, Shenzhen, China) has been described in detail elsewhere (Guo et al., 2021; Ge et al., 2022). In brief, it consists of an upper part (40/70 mm length), a waist (10 mm height) where the four branches arise, and a lower part that has a smaller diameter than the upper part (70 mm length), as shown in Figure 1A. The upper part has a diameter of 24–40 mm (4 mm increments) and the lower part is 14–18 mm in diameter (2 mm increments). The waist includes two inner branches for reconstruction of the CA and SMA and two outer branches for reconstruction of the two renal arteries. The inner branches have a length of 20/23 mm and diameter of 10 mm, and the outer branches measure 7 mm across and 15 mm in length (Figure 1A). The inner and outer branches are connected to the renovisceral arteries by self-expanding polytetrafluoroethylene-covered bridging stent grafts (BSGs; SilverFlowPV; Lifetech Scientific). The G-branch and BSGs composed of an “off-the-shelf” and multicomponent system together.

In this study, we investigated the morphological and hemodynamic performance of the G-Branch when used to repair a type III TAAA in a woman who was followed up for 12 months. Details of all the devices used are shown in Supplementary Table S1. Computed tomography angiography (CTA) scans of the chest, abdomen, and pelvis were obtained preoperatively, before discharge, and 6 and 12 months later. The CTA acquisition protocol varied slightly between the four scan times in order to obtain scans of the best possible quality.

The study protocol was approved by the Chinese PLA General Hospital ethics committee (reference: 2021-NO.-007). Written informed consent for publication of this report was obtained from the patient.

### 2.2 Assessment of morphology

Vascular straightening, also called “tubular flow straightening,” is a method by which curved vessels are stretched into a straight line along the vessel centerline. The CSA of vessels measured after straightening can eliminate the measurement error caused by the bending of vessels and achieve more accurate detection of lesions. 3mensio Vascular™ (3Mensio Medical Imaging, Bilthoven, Netherlands) can automatically segment and reconstruct 3D models of the blood vessel according to CTA images, semi-automatically generate the centerlines and straighten the blood vessel according to the center line to facilitate the measurement of CSA of blood vessels. In the measurement process, each renovisceral artery was semi-automatically generated a centerline, and the centerlines ranged from the vessel branch point or the initial sites of the BSGs to the bifurcation point. The vessel branch point was defined as the ostia of the renovisceral arteries from the abdominal aorta. The bifurcation point was defined as the first bifurcation on the renovisceral arteries (Suh et al., 2013). The branched point was defined as the terminals of the outer or inner branched stent grafts. Using the method described by Georgakarakos et al. (2014) and according to the following five points: (I) the initial site of the BSGs; (II) the branched point; (III) the vessel branch point; (IV) the distal end of the BSGs; (V) the bifurcation point, renovisceral arteries can be divided into up to four zones after the BSGs implantation. The branched zone between the initial sites of the BSGs and the branched point; the bridging zone between the branched point and the vessel branch point; the distal sealing zone between the vessel branch point and the distal end of the BSGs; and the native vessel zone between the distal end of the BSGs and the bifurcation point (Figure 1).

CSA was measured manually on the 3mensio workstation. CSA measurement of vessels covered by stents are shown in Figures 1D, E, and that of vessels not covered by stents or before operation are shown in Figure 1F. The centerline paths were then used to quantify the CSA at 5-mm intervals (Figure 1). All centerlines were matched and aligned between the pre- and post-intervention by two points, (II) the branched point and (V) the bifurcation point.

### 2.3 Model reconstruction and mesh generation

**FIGURE 2:**
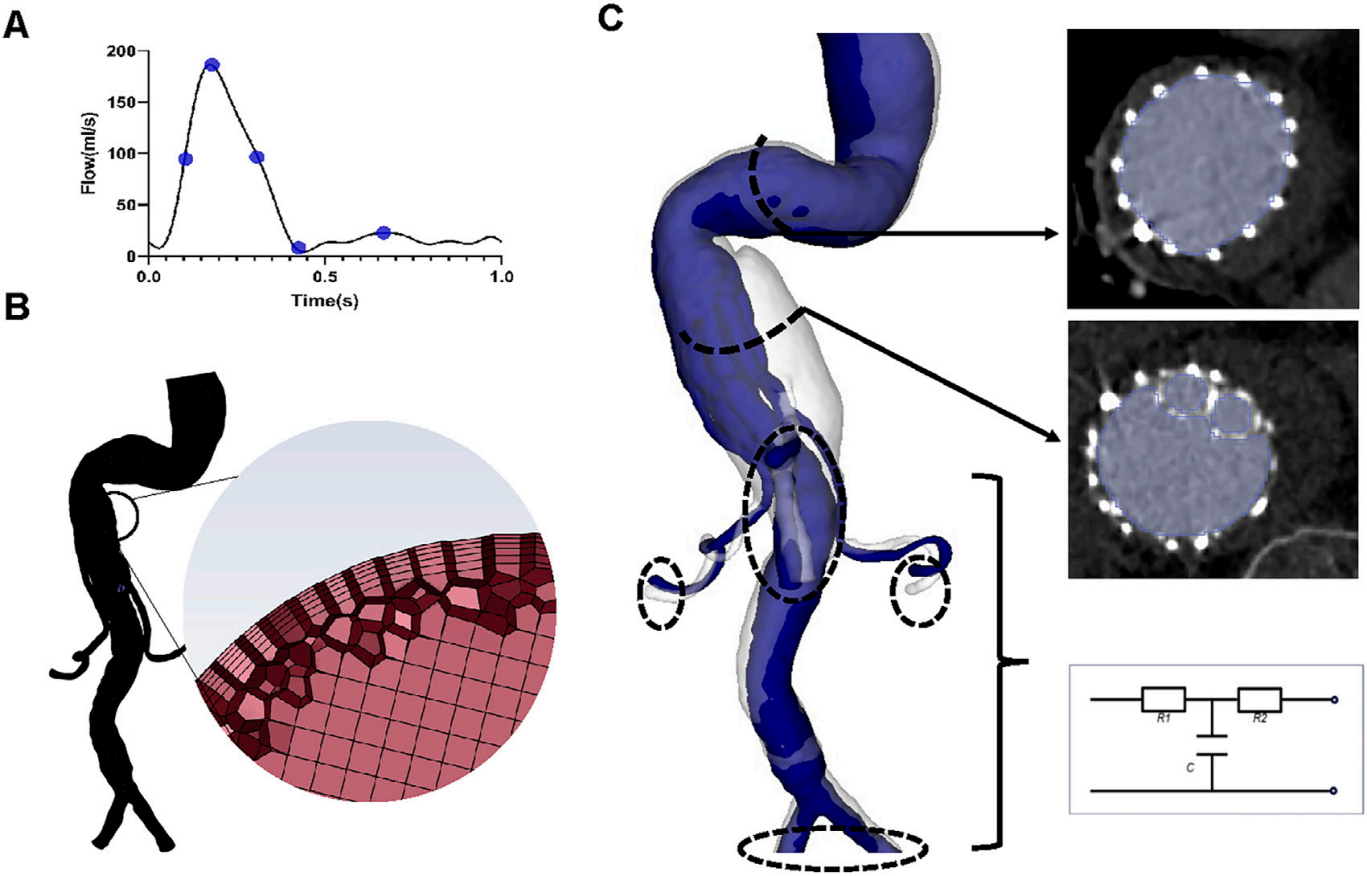
Reconstruction of the three-dimensional model, meshing, and boundary conditions. **(A)** The inlet flow rate waveform. Five specific time points, namely, systolic acceleration (T1 = 0.10 s), maximum flow rate (T2 = 0.18 s), systolic deceleration (T3 = 0.32 s), minimum flow rate (T4 = 0.44 s), and mid-diastole (T5 = 0.68 s), were chosen to depict hemodynamic changes over a cardiac cycle. **(B)** Sketches showing mesh generation. **(C)** Segmentation of the region of interest and three-element Windkessel model applied on each outlet.

The CTA images were also used to build three-dimensional models (Figure 2) using 3D Slicer software (version 4.11.20210226). The regions of interest were segmented manually (using a contouring method) and semi-automatically (using a thresholding method) (Figure 2). The regions of interest in G-branch endograft were depicted in Figure 2C, and that in renovisceral arteries were segmented according to Figures 1D–F. Next, a smoothing filter was applied to the three-dimensional models. The models were then optimized using Geomagic Studio software (3D Systems, Morrisville, NC, United States) before meshing (Figure 2).

A mesh dependency test was performed so that the relative error in two consecutive mesh refinements was less than 2% for the inlet maximum pressure of steady-state flow under the peak systolic condition. A total of 2,322,749–2,910,444 polyhedral-shaped volume elements were found to be adequate for accurate meshing of the computational domains. Five prism layers were created near the boundaries to improve the accuracy of model meshing (Figure 2).

### 2.4 Computational model

Blood flow patterns were determined by solving the Navier-Stokes and continuity equations using the Fluent finite volume solver (Ansys Inc., Canonsburg, PA, United States). The walls of the aorta and stent grafts were characterized by non-slip rigid wall boundary conditions (Kelsey et al., 2017). Although blood exists as a suspension of particles, it behaves as a Newtonian fluid in vessels with a diameter >1 mm (Wurfel, 1992). Moreover, we have found a negligible difference in hemodynamic parameters between Newtonian and non-Newtonian (i.e., Carreau fluid) models (Yin et al., 2016). Blood has been modelled as an incompressible Newtonian fluid with a density of 1,050 kg/m^3^ and a viscosity of 0.0035 Pa∙s (Kelsey et al., 2017; Mei et al., 2020).

A time-varying flow rate waveform derived from a study by Les et al. (2010) was used as the inlet boundary condition (Figure 2), for which the maximum Reynolds numbers of the inlet in each of the four geometric models varied between 1400 and 1529, while the Womersley number ranged from 24.41 to 26.65. Therefore, blood flow was considered laminar (Nerem et al., 1972).

The three-element Windkessel model (RCR circuit) was applied to each outlet (Figure 2) to approximate resistance and compliance in the vascular bed downstream. This method has been described in detail by Mei et al. (2020) (Supplementary Material). All Windkessel parameters were shown in Table 1.

**TABLE 1:** All Windkessl parameters applied on each outlet.

	Pre-operation			Pre-discharge			6 months			1 year		
	*Rp* (Pa•s•m^−3^)	*Rd* (Pa•s•m^−3^)	*C* (m^3^•Pa^−1^)	*Rp* (Pa•s•m^−3^)	*Rd* (Pa•s•m^−3^)	*C* (m^3^•Pa^−1^)	*Rp* (Pa•s•m^−3^)	*Rd* (Pa•s•m^−3^)	*C* (m^3^•Pa^−1^)	*Rp* (Pa•s•m^−3^)	*Rd* (Pa•s•m^−3^)	*C* (m^3^•Pa^−1^)
CA	1.19E + 08	1.20E + 09	1.50E−09	1.49E + 08	1.50E + 09	1.20E−09	1.33E + 08	1.34E + 09	1.35E−09	1.22E + 08	1.23E + 09	1.47E−09
SMA	8.44E + 07	8.50E + 08	2.12E−09	8.65E + 07	8.72E + 08	2.06E−09	9.77E + 07	9.84E + 08	1.83E−09	7.74E + 07	7.79E + 08	2.31E−09
LRA	2.93E + 08	2.96E + 09	6.08E−10	3.54E + 08	3.56E + 09	5.04E−10	2.90E + 08	2.93E + 09	6.15E−10	2.12E + 08	2.13E + 09	8.43E−10
RRA	1.88E + 08	1.90E + 09	9.48E−10	2.75E + 08	2.77E + 09	6.50E−10	2.64E + 08	2.66E + 09	6.76E−10	1.79E + 08	1.81E + 09	9.95E−10
LCIA	7.00E + 07	7.05E + 08	2.55E−09	6.39E + 07	6.43E + 08	2.80E−09	7.40E + 07	7.45E + 08	2.41E−09	6.15E + 07	6.19E + 08	2.91E−09
RCIA	9.13E + 07	9.20E + 08	1.96E−09	7.92E + 07	7.97E + 08	2.25E−09	8.68E + 07	8.74E + 08	2.06E−09	7.97E + 07	8.03E + 08	2.24E−09

CA, celiac artery; SMA, superior mesenteric artery; LRA, left renal artery; RRA, right renal artery; LCIA, left common iliac artery; RCIA, left common iliac artery; Rp, proximal resistance; Rd, distal resistance; C, vessel compliance.

Each simulation ran for three cardiac cycles with a time step of 0.01 s, and scaled residuals to 10^−5^ were imposed as the convergence criterion. The results of the third cycle were used for the analysis (Fan et al., 2016). The postoperative performance of the G-Branch was assessed in terms of the flow pattern, including flow rates, velocity and pressure, and hemodynamic parameters. The hemodynamic parameters, which included the time-averaged wall shear stress (TAWSS), oscillatory index (OSI), relative resident time (RRT), SAR-TAWSS, SAR-OSI, and SAR-RRT (Supplementary Table S2), were determined from the computed flow fields (Fan et al., 2016). SAR-TAWSS was referred as the percentage of surface area with low TAWSS (<0.4) to the total area. SAR-OSI was referred as the percentage of surface area with high OSI (>0.3) to the total area. And SAR-RRT was referred as the percentage of surface area with high RRT (>10) to the total area.

### 2.5 Statistical analysis

Continuous variables are presented as the mean ± standard deviation if normally distributed and as the median (interquartile range) if not. All statistical analyses were performed using GraphPad Prism (GraphPad Software, San Diego, CA, United States). D’Agostino-Pearson tests were applied to determine normality. Statistical significance was examined using the Kruskal–Wallis test with Dunn’s multiple comparison test (Northrup et al., 2022). *p*-values were adjusted automatically by GraphPad Prism and considered statistically significant when <0.05.

## 3 Results

### 3.1 Morphology

**FIGURE 3:**
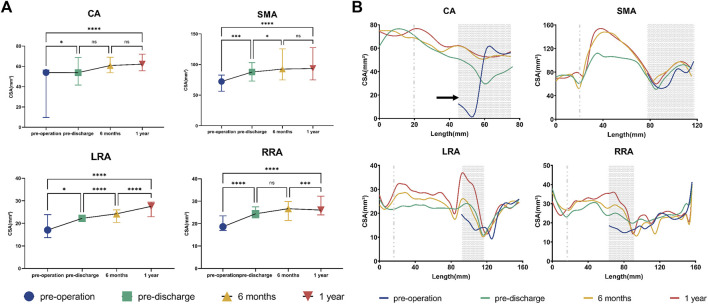
Cross-sectional area of bridging stent grafts and renovisceral arteries. **(A)** Cross-sectional area of the renovisceral arteries. The symbols show the median and the error bars showed the interquartile range. *p < 0.05; ***p 0.001; ****p < 0.0001; ns, not statistically significant. **(B)** Cross-sectional area by distance from the initial site of the bridging stent graft in 5-mm increments. The initial site of the bridging stent graft is assigned to zero on the x-axis. The distal sealing zones are shown as gray areas. The gray dotted line indicates the branched point. The areas on the left of the gray dotted line indicate the branched zones. The regions between the gray dotted line and the gray area represent the bridging zones, while the native vessel zones occupy the areas to the right of the gray areas. The black circles indicate stenosis in the vessels or stent grafts. CA, celiac artery; SMA, superior mesenteric artery; LRA, left renal artery; RRA, right renal artery; LCIA, left common iliac artery; RCIA, right common iliac artery.

The median CSA of all BSGs showed a consistent upward trend during follow-up (Figure 3). Quantitative analysis identified statistically significant differences in the CSA of all BSGs between before and after surgery [CA, *p* < 0.05; SMA, *p* < 0.001; left renal artery (LRA), *p* < 0.05; right renal artery (RRA), *p* < 0.0001]. However, there was no significant difference in the CSA of either the CA or SMA post-discharge. Findings were similar for the CSA of the G-Branch (Supplementary Figure S1). However, differences in the CSA of both renal arteries were statistically significant at almost all time points before and after surgery (Figure 3).

The BSGs in the CA and SMA were extended into the bifurcation point and divided into three zones and those in the two renal arteries were divided into four zones (Figure 3). During follow-up, there were no obvious changes in CSA in the branched zones of any of the BSGs, in the distal sealing zone of the SMA, or in the native vessel zone of either renal artery. The CSA of the bridging zones in all BSGs increased gradually during follow-up, particularly in the SMA and LRA. The CSA tended to increase in the distal sealing zones of the CA and both renal arteries (Figure 3). Moreover, a typical “J” shape was found in the CA, indicating severe proximal stenosis caused by compression of the median arcuate ligament (Sugae et al., 2012) (Figure 3; Supplementary Figure S2), which was relieved during follow-up after BSG implantation.

### 3.2 Flow rate

**FIGURE 4:**
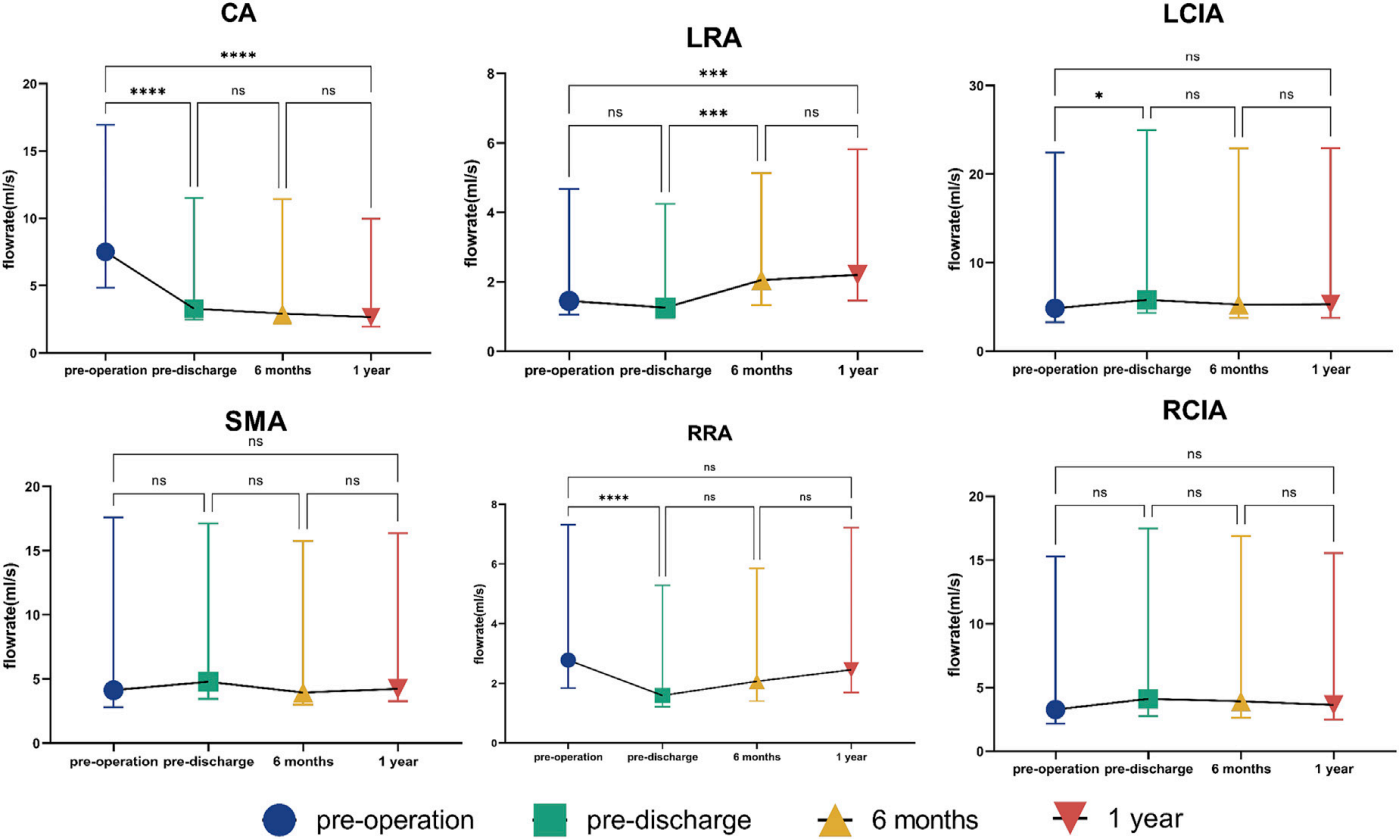
Flow rate in outlets. The symbols show the median and the error bars indicate the interquartile range. *p < 0.05; ***p < 0.001; ****p < 0.0001; ns, not statistically significant. CA, celiac artery; SMA, superior mesenteric artery; LRA, left renal artery; RRA, right renal artery; LCIA, left common iliac artery; RCIA, right common iliac artery.

The change in flow distribution is important when evaluating the effect of endograft implantation. There was no statistically significant difference in flow rate in either of the common iliac arteries or in the SMA at almost all time points before and after surgery. There was no significant change in flow rate in any of the vessels between 6 and 12 months (Figure 4). The change in flow rate in the CA before and after surgery was statistically significant but not during follow-up. Overall, there was a reduction in flow rate in the CA of about 30% after surgery. However, the flow rate in the LRA increased over time, finally reaching an increase of nearly 30%. The flow rate in the RRA showed a “down-up” trend, with no statistically significant difference between the value before surgery and that 12 months later (Figure 4). Supplementary Figure S3 shows the details of the flow rate waveforms for all outlets. Changes of flow rate was present at the time of systolic deceleration and diastole over a cardiac cycle in the CA, LRA, and RRA.

### 3.3 Flow patterns and pressure

**FIGURE 5:**
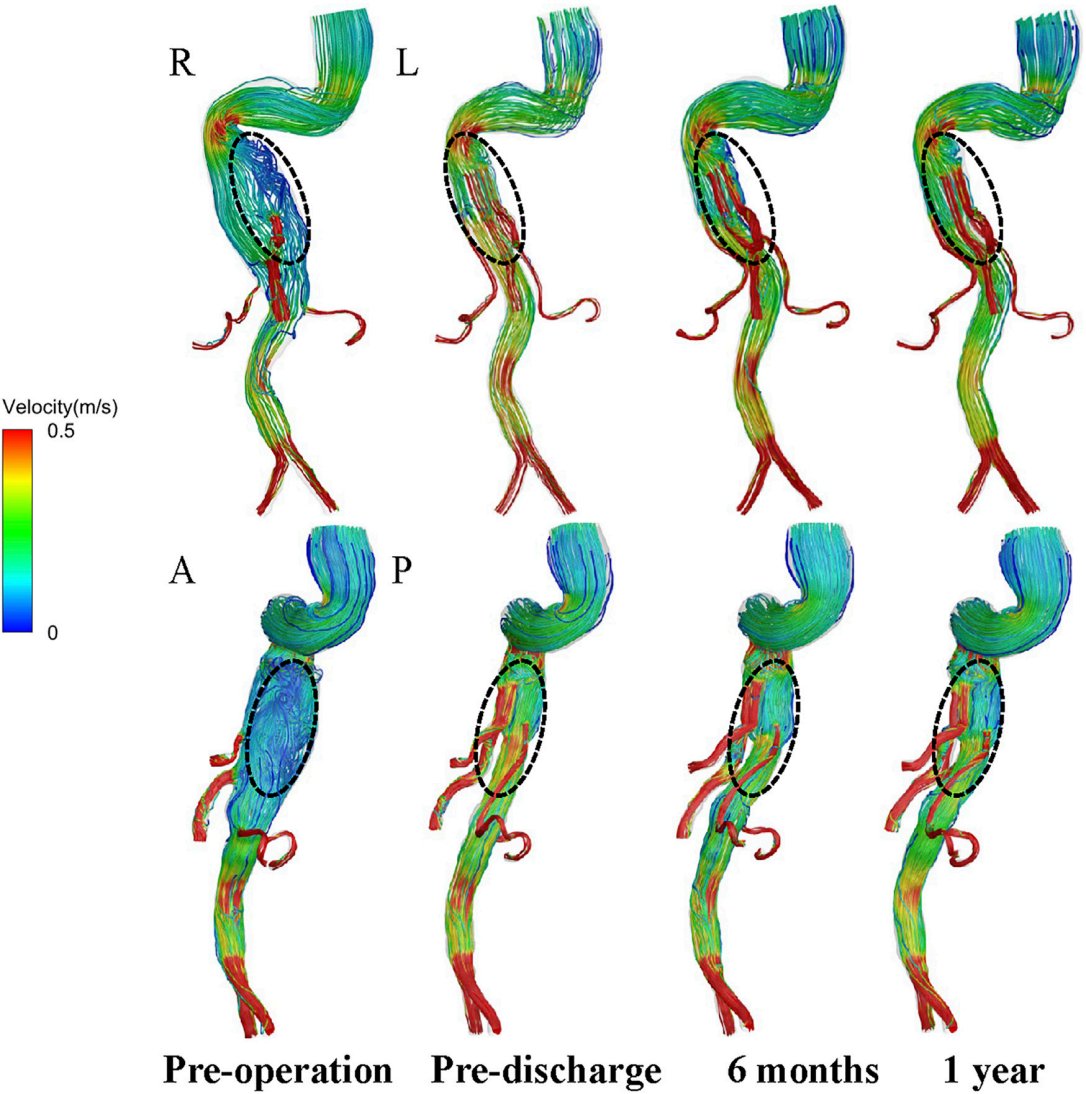
Velocity path lines in all models (t = 0.18 s, maximum flow rate). A, P, L and R denote the locations of the anterior, posterior, left and right walls, respectively.

Instantaneous velocity streamlines before surgery, before discharge, and at 6 and 12 months postoperatively were compared at the time points of maximum flow rate (t = 0.18 s) (Figure 5). The flow patterns in the aortic aneurysm changed after implantation of the G-Branch and remained stable after surgery. Large eddies and spiral flow were generated in the aortic aneurysm at peak systole, which converted to undisturbed laminar flow in the G-Branch endograft. Flow patterns in the BSGs were relatively consistent after surgery. Instantaneous velocity magnitudes in different cross-sectional planes at the maximum flow rate (t = 0.18 s) in the cardiac cycle are shown in Supplementary Figure S4.

**FIGURE 6:**
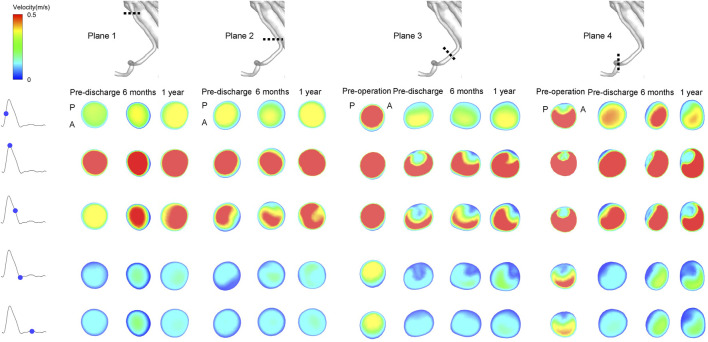
Instantaneous velocity magnitudes of different cross-sectional planes in the right renal artery at five phases of the cardiac cycle. Plane 1 is from the branched zone; Plane 2 is from the bridging zone; Plane 3 is from the distal sealing zone; Plane 4 is from the native vessel zone. A and P denote the locations of the anterior and posterior walls, respectively.

**FIGURE 7:**
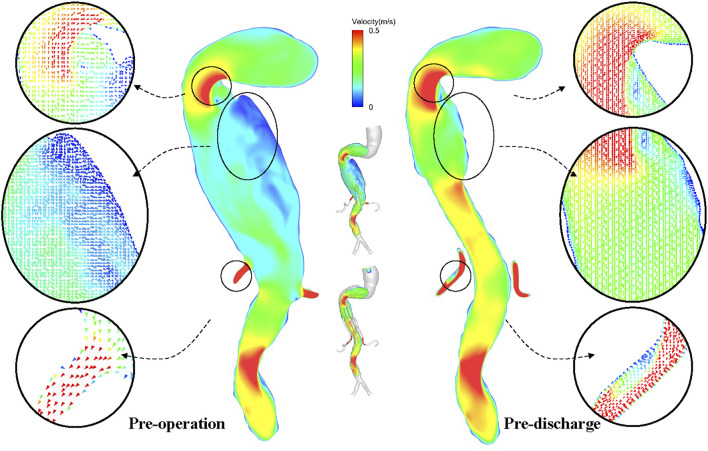
Detailed velocity vector before and after surgery.

Instantaneous velocity contours in four specific planes taken from the RRA are shown in Figure 6. Plane 1 is from the branched zone, Plane 2 is from the bridging zone, Plane 3 is from the distal sealing zone, and Plane 4 is from the native vessel zone. There was no obvious swirling in Planes 1 and 2 of the RRA but zones of stasis were observed, and this flow pattern did not change dramatically during follow-up. However, in Plane 3 of the RRA, eddies started to appear at the time of maximum flow rate postoperatively and continued during follow-up. Eddies and zones of stasis were also observed in Plane 4 of the RRA. Instantaneous velocity contours in the CA, SMA, and LRA are shown in Supplementary Figures S5–S7. Eddies were also present in the bridging zones of the CA and SMA and in the distal sealing zone of the LRA. Details of the velocity vector before and after surgery are presented in Figure 7.

Supplementary Figure S8 shows the pressure distribution along the G-Branch and BSGs, which was significantly decreased at pre-discharge compared with before surgery and remained stable at 6 and 12 months after surgery. The decrease in time-averaged pressure between the inlet and each outlet is shown in Supplementary Figure S8. The most dramatic pressure decrease was in the CA BSG.

### 3.4 Other hemodynamic parameters

**FIGURE 8:**
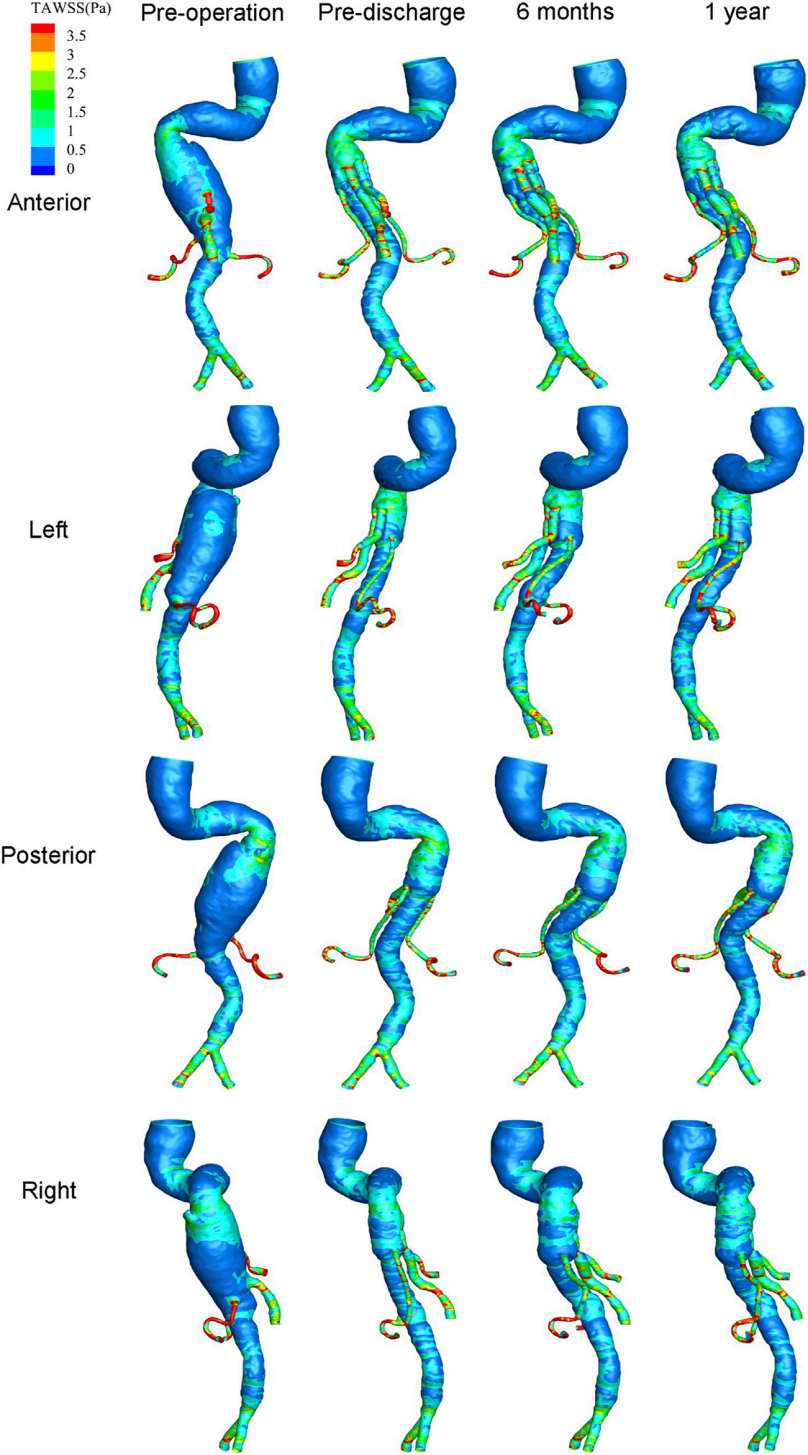
Distribution of time-averaged wall shear stress at four positions before and after surgery.

**FIGURE 9:**
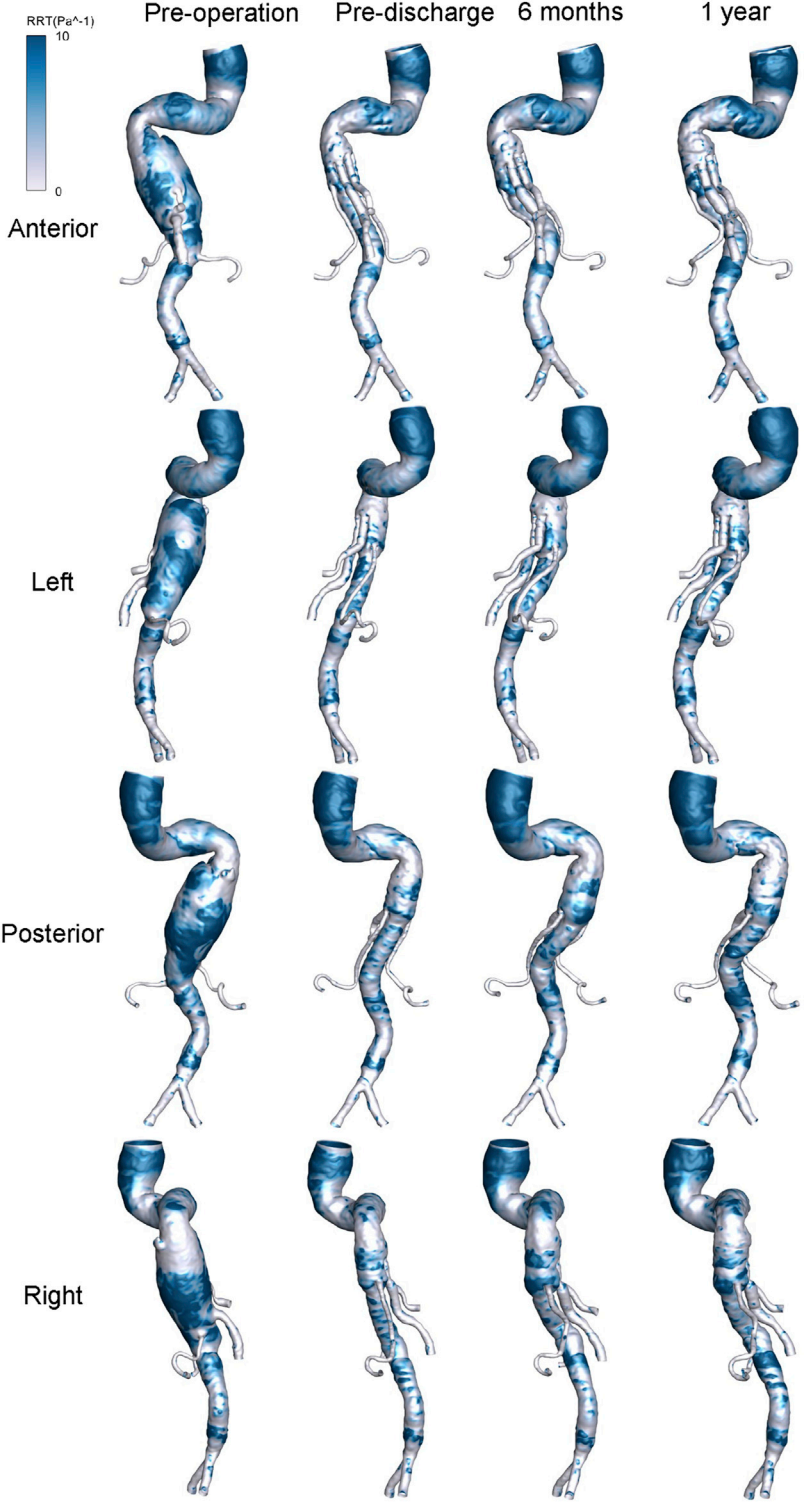
Distribution of relative resident time at four positions before and after surgery.

The distribution of hemodynamic parameters in the wall is closely related to vascular remodeling. The corresponding distributions of TAWSS (Pa) and RRT (Pa^−1^) are shown in Figure 8 and Figure 9, respectively. Figure 8 shows the distribution of TAWSS before and after surgery. Before surgery, TAWSS was low throughout the entire aorta. After G-Branch implantation, TAWSS in the G-Branch segment was higher than that either upstream or downstream of the endograft. Furthermore, TAWSS was higher in the renovisceral arteries and BSGs preoperatively and postoperatively. Supplementary Figure S9 shows the distribution of the OSI before and after surgery.

The risk of thrombosis is an important consideration when evaluating the quality of surgery for an aneurysm, for which RRT is an accepted parameter. Figure 9 shows the distribution of RRT on the wall. It is worth noting that the regions of low TAWSS, high OSI, and high RRT almost overlapped. The high RRT distribution disappeared in a large area of the aneurysmal sac after G-branch implantation, whereas the RRT distribution in the renovisceral arteries and BSGs was consistently low before and after surgery.

**FIGURE 10:**
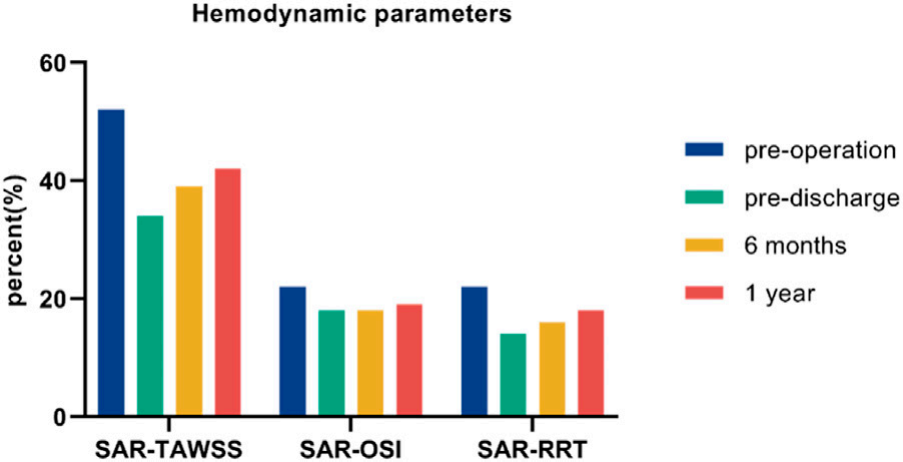
Scalar hemodynamic parameters before and after surgery.

In view of the complex distribution of TAWSS, OSI, and RRT, we used scalar parameters for SAR-TAWSS, SAR-OSI, and SAR-RRT to enhance the quantitative representation of the hemodynamic environment (Figure 10). SAR-TAWSS and SAR-RRT decreased markedly after G-Branch implantation.

## 4 Discussion

Total endovascular aortic repair is being used increasingly in patients with TAAAs. The G-Branch endograft is a promising device for TAAAs repair in view of its conformation, which includes two inner branches and two outer branches for reconstruction of the renovisceral arteries. Based on the three-dimensional geometry reconstructed from CTA images, we computed the flow field of the G-Branch in a patient with the TAAA over time. Our research had two main findings. First, the G-Branch successfully excluded the aneurysmal sac, the BSGs reconstructed the renovisceral arteries, and the CSA in the G-Branch and BSGs maintained an upward trend during follow-up. Second, after surgery, there was a redistribution of flow rate with an increase in velocity, an increase in TAWSS, and a decrease in OSI and RRT in the G-Branch and BSGs.

Morphological changes have been investigated in fenestrated and chimney stent grafts (Ullery et al., 2015; Tricarico et al., 2017), and several parameters have been suggested for depiction of the anatomic structure in three-dimensional models. One is curvature, which describes how rapidly a curve deviates from a straight line, the lower the magnitude and the more gradual the curvature. Unfortunately, curvature may not be able to predict occlusion or stenosis of a stent graft (Sylvan et al., 2016). The other parameter is CSA, which has a significant relationship with occlusion or stenosis of a stent graft. In the study by Tricarico et al. (2017), a CSA of <14 mm^2^ was significantly associated with stent graft occlusion following chimney endovascular repair of juxtarenal aortic aneurysms. Chimney stent grafts seem to be compressed in almost all renovisceral arteries, despite use of covered balloon-expandable stents.

Morphological remodeling occurs between the aortic wall and stent grafts after implantation of the G-Branch and BSGs. In our study, the CSA increased gradually over time after implantation of this multibranched endograft with self-expandable covered BSGs, despite the involvement of only one investigator (Figure 3). Expansion of the CSA was sustained particularly in the bridging zones of all renovisceral arteries and the distal sealing zones of the CA and both renal arteries. Stable morphology was found in the branched zones of all renovisceral arteries and native vessel zones of the two renal arteries. Segments of both renal arteries with a CSA of <14 mm^2^ were located at the junction of the distal sealing and native vessel zones. However, no obvious stenosis or occlusion occurred during 12 months of follow-up. Given that previous studies have focused on morphological changes with fenestrated and chimney stent grafts (Ullery et al., 2015; Tricarico et al., 2017), whether a large curvature or small CSA of BSGs in a multibranched device could herald a poor prognosis needs future investigation. Self-expandable stent grafts may be alternative devices for treatment of TAAA in patients with compression of the CA (Figure 3; Supplementary Figure S2).

Reconstruction of the renovisceral arteries and preservation of blood flow are important in the treatment of TAAA. In our study, a decreased flow rate did not lead to deterioration of physiological function. In the absence of patient-specific inlet flow waves, a uniform time-averaged volumetric flow rate of 2.8 L/min was applied at the thoracic aortic inlet in all four three-dimensional models (Figure 2) and was within the physiological range (Critchley, 1999). We then investigated the division of blood flow in the renovisceral arteries and both common iliac arteries and found no changes in the 12 months following G-Branch implantation (Figure 4). Furthermore, blood was redistributed in the renovisceral arteries. The flow rate in the CA was reduced by approximately 30% post-surgery and the blood was subsequently transferred to the LRA. Blood flow in the renal arteries did not appear to have decreased dramatically by G-Branch implantation. We also found that a marked reduction in CSA did not lead to a decrease in flow rate, particularly in the LRA, during 12 months of follow-up. Furthermore, in clinical practice, Alanine and Aspartate aminotransferases are classical biomarkers indicating liver parenchyma damage. Actually, we found that the value was decreasing after operation, which indicated that the change in flow rate of CA did not have a catastrophic impact on liver parenchyma (Supplementary Table S3).

The conformation of the G-Branch seems reasonable. Changes in flow patterns after surgery were evaluated by instantaneous velocity streamlines and cross-sectional velocity contours. First, the large eddies and zones of stasis in the aneurysmal sac before surgery disappeared after G-Branch implantation (Figure 5; Supplementary Figure S4). Meanwhile, with the exception of the segment covered by the endograft, flow upstream and downstream of the aneurysmal sac was barely affected by the intervention, which is consistent with the findings of Zhu et al. (2019). In their study, an endograft with two inner branches could successfully exclude an aneurysmal sac in the aortic arch from blood flow. Second, although a small FRZ persisted postoperatively because of the tortuosity of the proximal descending aorta, the inner branches did not have an apparent impact on flow patterns, given that blood still flowed smoothly into the CA and SMA through these branches. Several studies have demonstrated that FRZs and zones of stasis are at increased risk of thrombosis (Bluestein et al., 1996; Rayz et al., 2008; Jackson et al., 2009). Disappearance of flow recirculation and stasis zones demonstrates the feasibility of the G-Branch, but with regard to the BSGs, the bridging zones in the CA and SMA as well as the distal sealing zones in both renal arteries warrant more attention. Large flow recirculation areas were recognized in these zones.

It is known that hemodynamic parameters (i.e., WSS < 0.4 Pa and OSI > 0.3) contribute to endothelial cell function and gene expression and are associated with vascular remodeling (Malek, 1999; Reneman et al., 2006). We found that large flow vortices within the aneurysmal sac before surgery resulted in low TAWSS, high OSI, and high RRT, which may lead to intraluminal thrombosis, progression of the aneurysm, and even rupture (Parker et al., 2019). Owing to the increasing velocities in the G-Branch and BSGs, TAWSS in the post-intervention aorta was much higher than in the pre-intervention model. Moreover, the aneurysm did not progress during follow-up. The hemodynamic assessments presented by SAR-TAWSS, SAR-OSI, and SAR-RRT were quantitative. As shown in Figure 10, there were marked decreases in SAR-TAWSS and SAR-RRT after G-Branch implantation. The same trend was seen regarding the surface area ratio of the high OSI. Elevated TAWSS and lower OSI and RRT in the G-Branch and BSGs reduced the likelihood of thrombosis and progression of the aneurysm. Instead, the TAWSS was lower and the OSI and RRT were higher proximal to the TAAA, and our patient received another treatment to repair this pathology. Importantly, the aneurysmal sac was successfully excluded in this patient after G-Branch implantation; accordingly, the improved and stable hemodynamic environment in the G-Branch resulted in an elevated TAWSS and a decrease in OSI and RRT. Compared with the distribution of hemodynamic parameters in the G-Branch, the TAWSS was relatively higher and the OSI and RRT were lower in all renovisceral arteries and BSGs before and after surgery.

This study has several limitations. First, it included only one patient in whom TAAA was repaired using a G-Branch endograft. While the findings were relatively robust, this small sample precludes any conclusions. Second, the three-dimensional model walls were assumed to be non-deformable and rigid rather than compliant. Moreover, movement between the endograft and the renal arteries during the cardiac cycle and respiration were not considered. Given that the patient had undergone repair with a thoracic endograft and a G-Branch endograft, the extensive coverage of the aorta made the wall relatively rigid and immobile. Fluid-structure interaction will be analyzed in our future work to improve the accuracy of our results.

## 5 Conclusion

After implantation of the G-Branch device and BSGs, CSA maintained an upward trend during the follow-up period. Furthermore, a redistributed flow rate, increased velocity, elevated TAWSS, and lower OSI and RRT were found in the G-Branch and BSGs. Branch vessels were revascularized after implantation of the G-Branch, and the improvements originated from not only changes in morphology but also in hemodynamics. The long-term performance of the G-Branch endograft needs further investigation and clinical validation.

## Data Availability

The original contributions presented in the study are included in the article/Supplementary Material, further inquiries can be directed to the corresponding author.

## Abbreviations

TAAAs, Thoracoabdominal aortic aneurysms; CFD, Computational fluid dynamics; BSGs, Bridging stent grafts; CA, Celiac artery; SMA, Superior mesenteric artery; RA, Renal artery; LRA, Left renal artery; RRA, Right renal artery; CIA, Common iliac artery; CTA, Computed tomographic angiography; CSA, Cross-sectional area; TAWSS, Time-averaged wall shear stress; OSI, Oscillatory index; RRT, Relative resident time; FRZs, Flow recirculation zones; MAL, Median arcuate ligament; MALS, Median arcuate ligament syndrome.
