# Supplementary material for: Aortic hemodynamic and morphological analysis before and after repair of thoracoabdominal aortic aneurysm using a G-Branch endograft
Source: Front Physiol. 2023 Aug 4;14:1234989. doi: 10.3389/fphys.2023.1234989 (PMC10438984; doi:10.3389/fphys.2023.1234989)
Supplement: Supplementary file 1 [file Presentation1.pptx]

## Slide 1
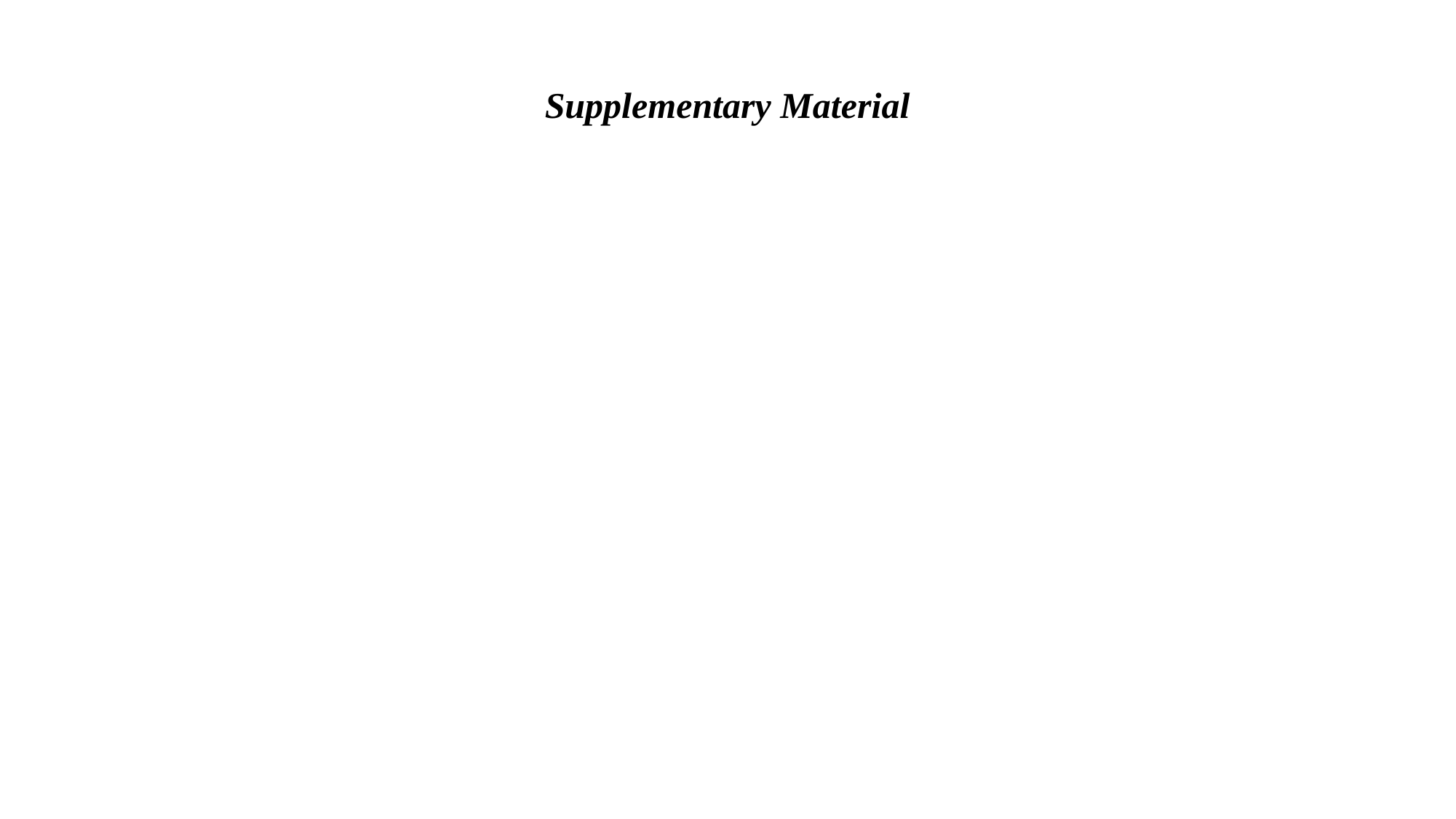

Supplementary Material

## Slide 2
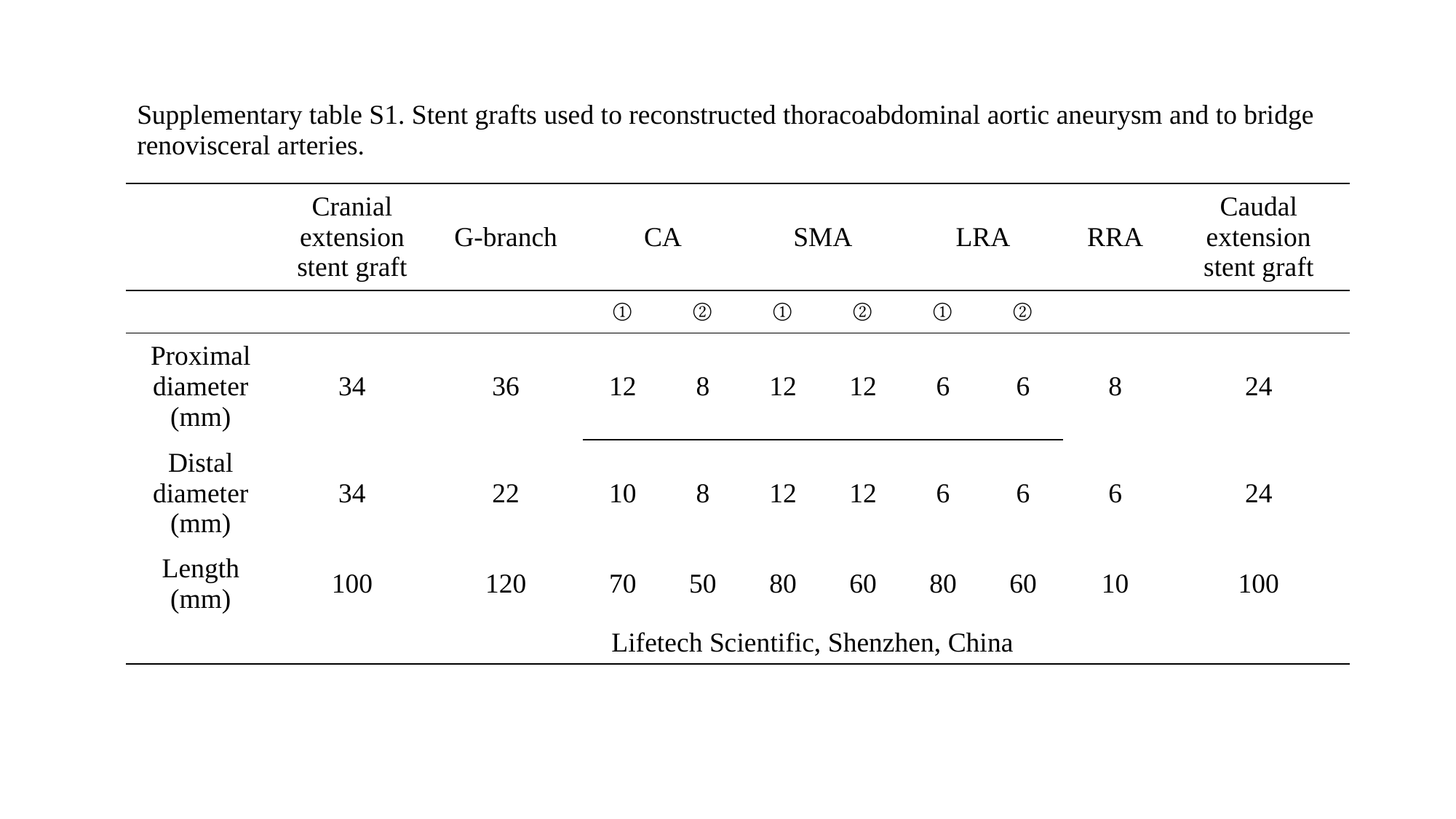

| Supplementary table S1. Stent grafts used to reconstructed thoracoabdominal aortic aneurysm and to bridge renovisceral arteries. | | | | | | | | | | |
| --- | --- | --- | --- | --- | --- | --- | --- | --- | --- | --- |
| | Cranial extension stent graft | G-branch | CA | | SMA | | LRA | | RRA | Caudal extension stent graft |
| | | | ① | ② | ① | ② | ① | ② | | |
| Proximal diameter (mm) | 34 | 36 | 12 | 8 | 12 | 12 | 6 | 6 | 8 | 24 |
| Distal diameter (mm) | 34 | 22 | 10 | 8 | 12 | 12 | 6 | 6 | 6 | 24 |
| Length (mm) | 100 | 120 | 70 | 50 | 80 | 60 | 80 | 60 | 10 | 100 |
| | Lifetech Scientific, Shenzhen, China | | | | | | | | | |

## Slide 3
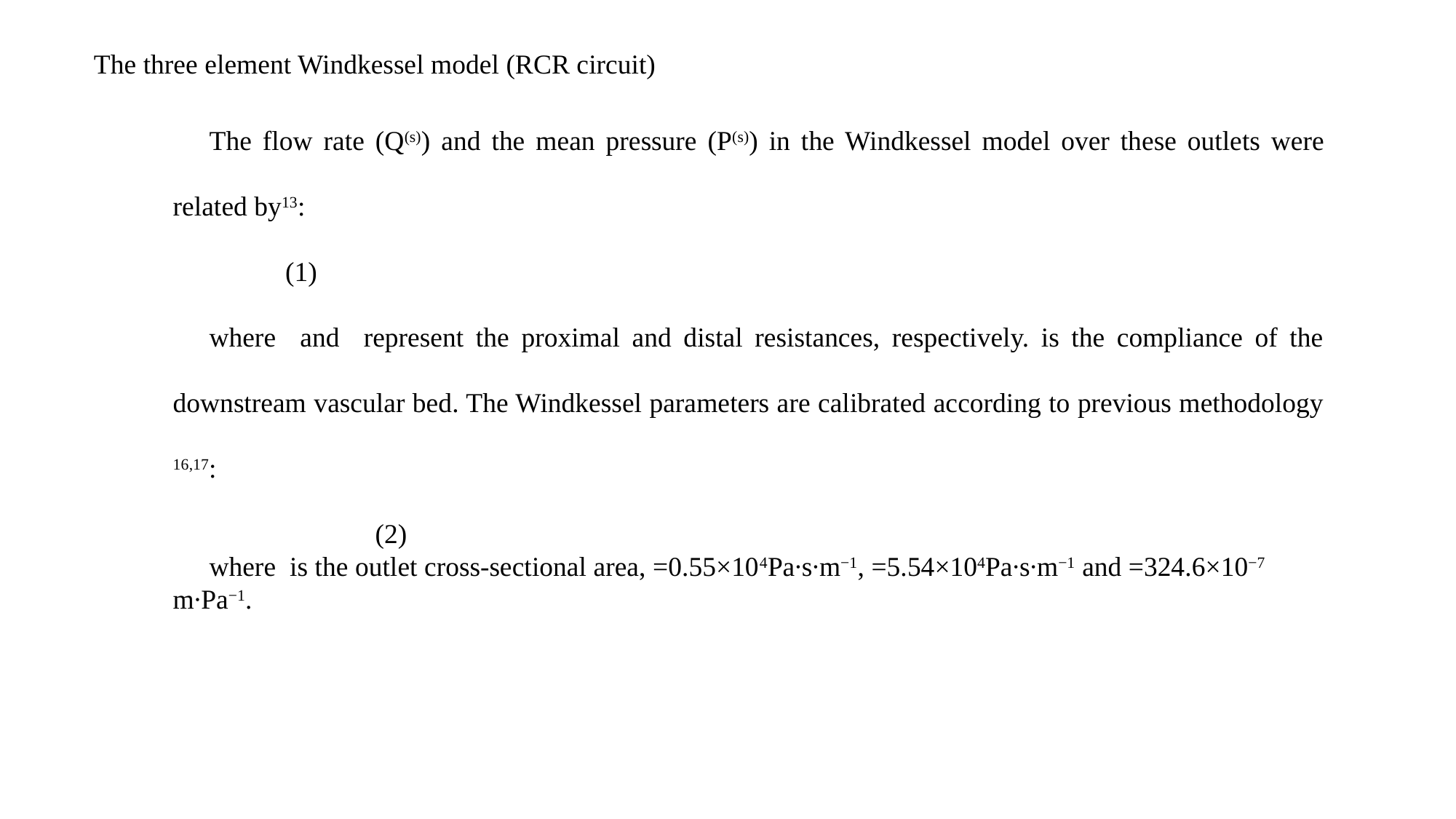

The three element Windkessel model (RCR circuit)

## Slide 4
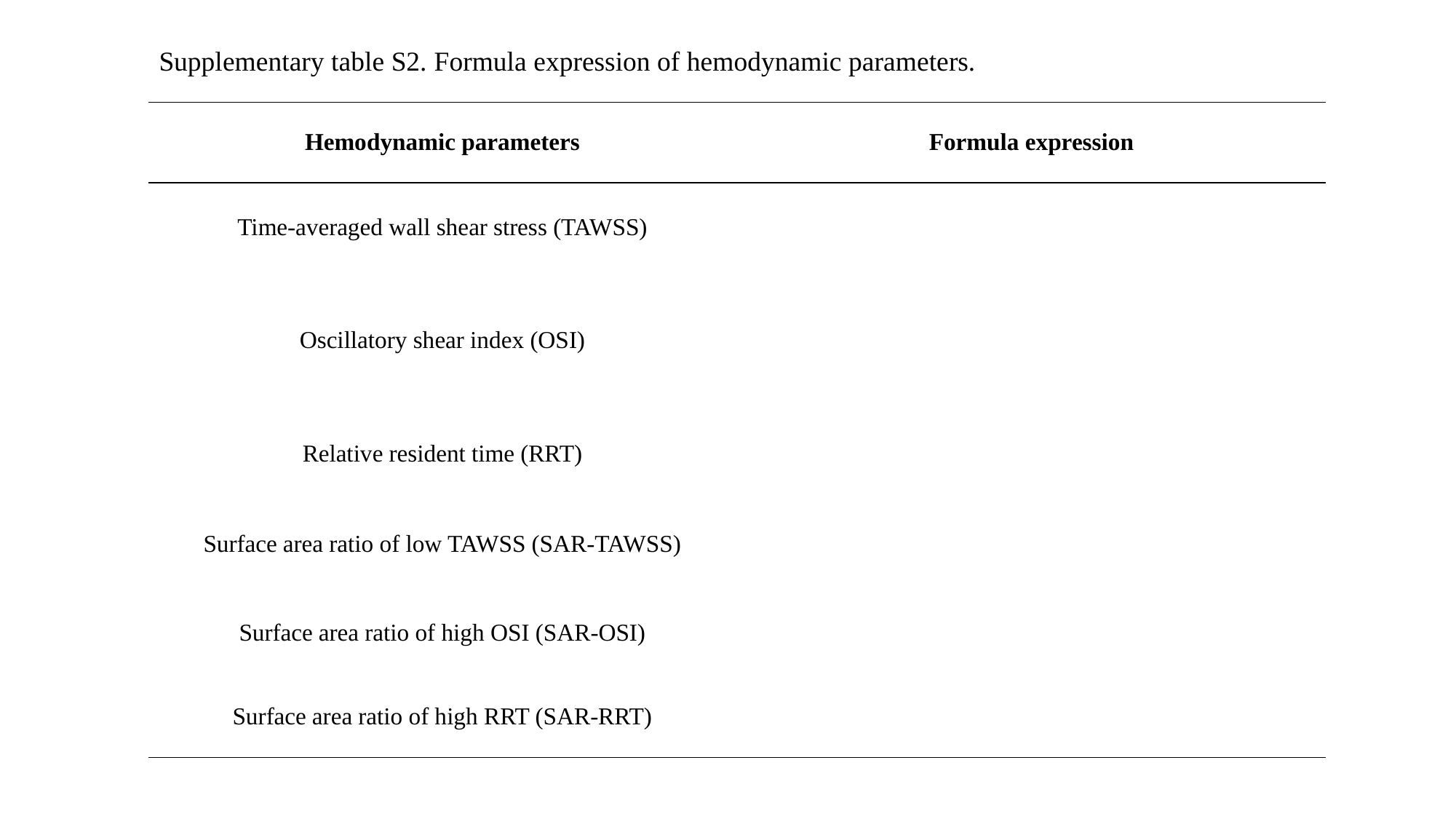

## Slide 5
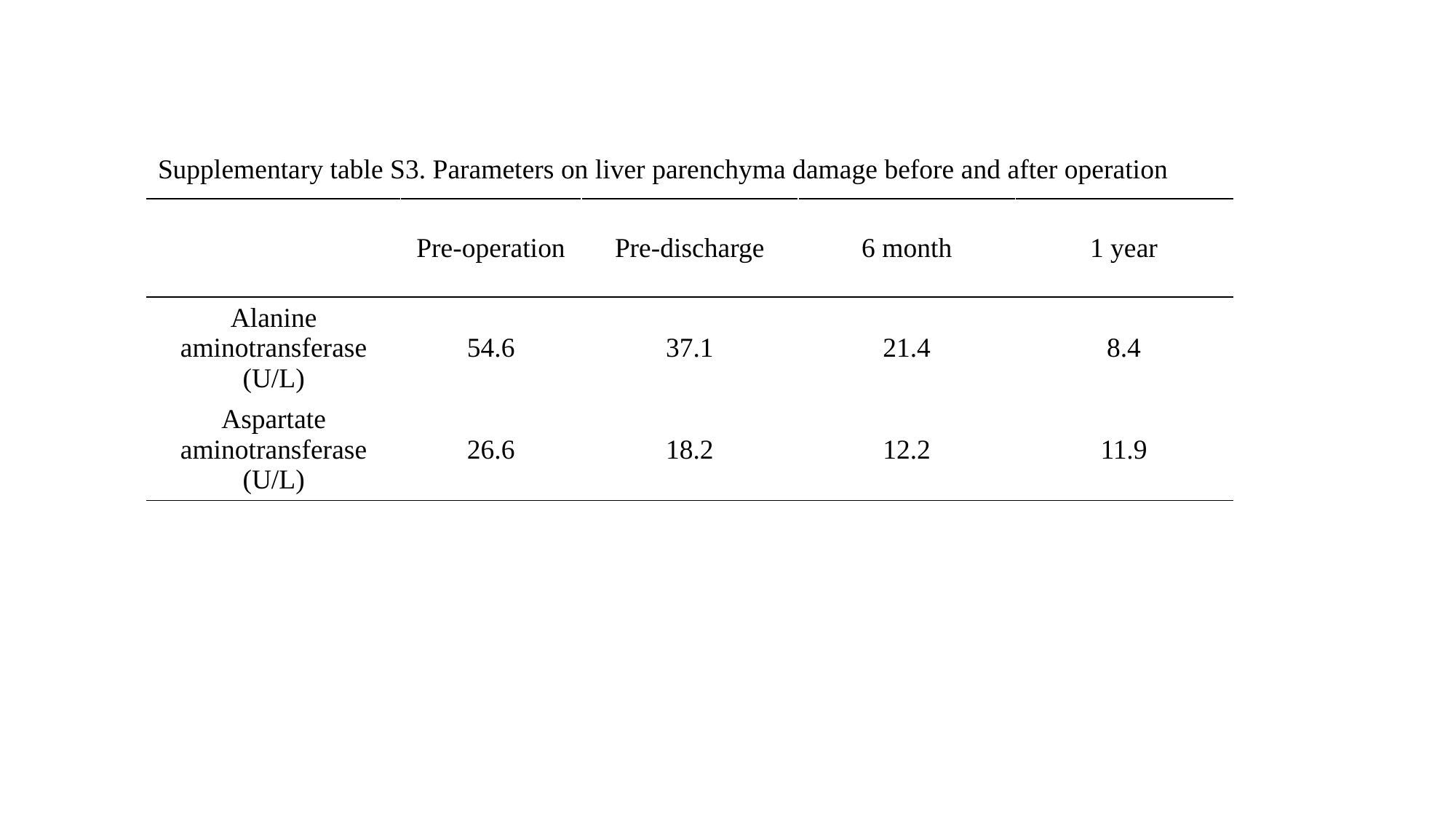

| Supplementary table S3. Parameters on liver parenchyma damage before and after operation | | | | |
| --- | --- | --- | --- | --- |
| | Pre-operation | Pre-discharge | 6 month | 1 year |
| Alanine aminotransferase (U/L) | 54.6 | 37.1 | 21.4 | 8.4 |
| Aspartate aminotransferase (U/L) | 26.6 | 18.2 | 12.2 | 11.9 |

## Slide 6
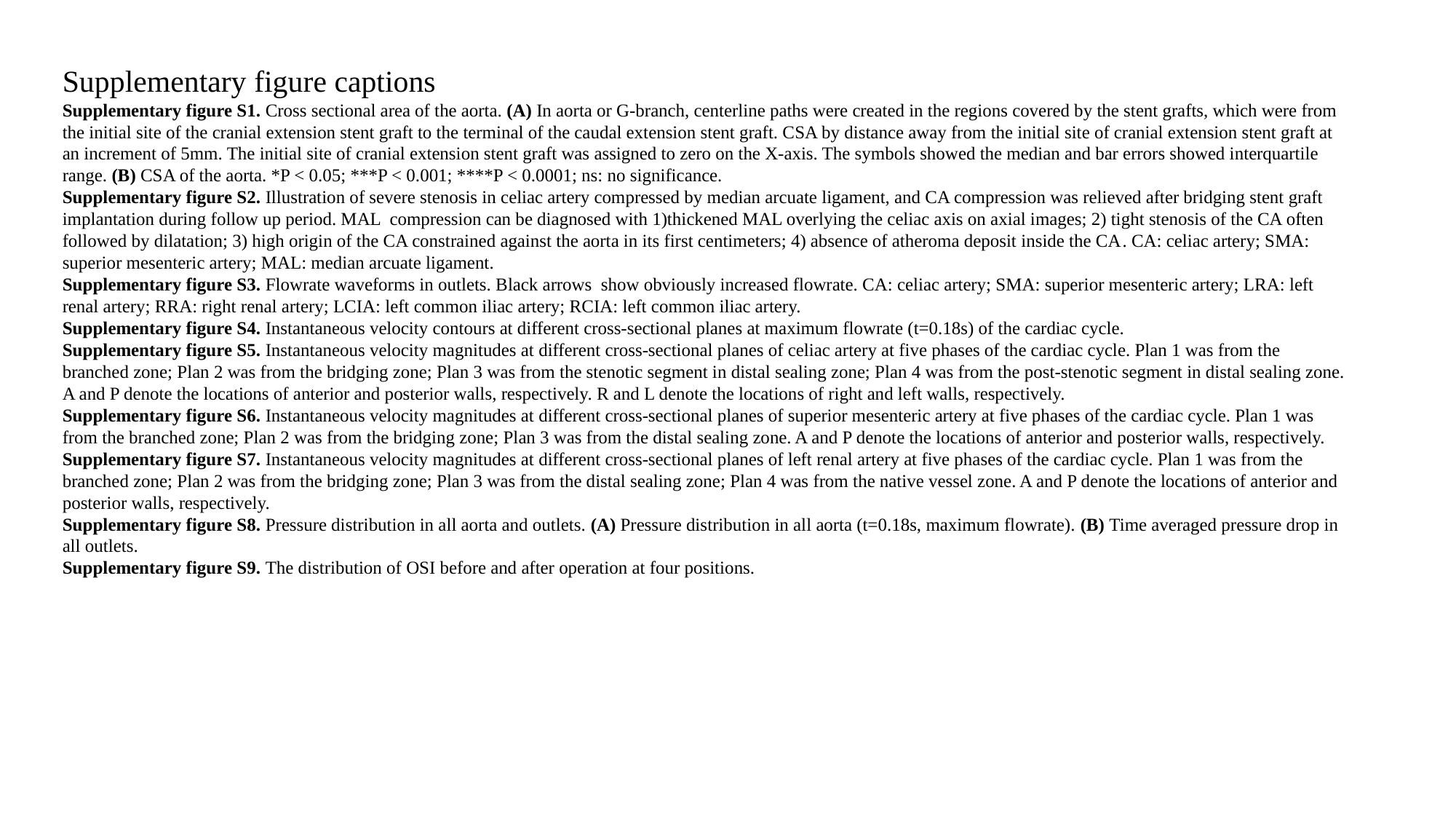

Supplementary figure captions
Supplementary figure S1. Cross sectional area of the aorta. (A) In aorta or G-branch, centerline paths were created in the regions covered by the stent grafts, which were from the initial site of the cranial extension stent graft to the terminal of the caudal extension stent graft. CSA by distance away from the initial site of cranial extension stent graft at an increment of 5mm. The initial site of cranial extension stent graft was assigned to zero on the X-axis. The symbols showed the median and bar errors showed interquartile range. (B) CSA of the aorta. *P < 0.05; ***P < 0.001; ****P < 0.0001; ns: no significance.
Supplementary figure S2. Illustration of severe stenosis in celiac artery compressed by median arcuate ligament, and CA compression was relieved after bridging stent graft implantation during follow up period. MAL compression can be diagnosed with 1)thickened MAL overlying the celiac axis on axial images; 2) tight stenosis of the CA often followed by dilatation; 3) high origin of the CA constrained against the aorta in its first centimeters; 4) absence of atheroma deposit inside the CA. CA: celiac artery; SMA: superior mesenteric artery; MAL: median arcuate ligament.
Supplementary figure S3. Flowrate waveforms in outlets. Black arrows show obviously increased flowrate. CA: celiac artery; SMA: superior mesenteric artery; LRA: left renal artery; RRA: right renal artery; LCIA: left common iliac artery; RCIA: left common iliac artery.
Supplementary figure S4. Instantaneous velocity contours at different cross-sectional planes at maximum flowrate (t=0.18s) of the cardiac cycle.
Supplementary figure S5. Instantaneous velocity magnitudes at different cross-sectional planes of celiac artery at five phases of the cardiac cycle. Plan 1 was from the branched zone; Plan 2 was from the bridging zone; Plan 3 was from the stenotic segment in distal sealing zone; Plan 4 was from the post-stenotic segment in distal sealing zone. A and P denote the locations of anterior and posterior walls, respectively. R and L denote the locations of right and left walls, respectively.
Supplementary figure S6. Instantaneous velocity magnitudes at different cross-sectional planes of superior mesenteric artery at five phases of the cardiac cycle. Plan 1 was from the branched zone; Plan 2 was from the bridging zone; Plan 3 was from the distal sealing zone. A and P denote the locations of anterior and posterior walls, respectively.
Supplementary figure S7. Instantaneous velocity magnitudes at different cross-sectional planes of left renal artery at five phases of the cardiac cycle. Plan 1 was from the branched zone; Plan 2 was from the bridging zone; Plan 3 was from the distal sealing zone; Plan 4 was from the native vessel zone. A and P denote the locations of anterior and posterior walls, respectively.
Supplementary figure S8. Pressure distribution in all aorta and outlets. (A) Pressure distribution in all aorta (t=0.18s, maximum flowrate). (B) Time averaged pressure drop in all outlets.
Supplementary figure S9. The distribution of OSI before and after operation at four positions.

## Slide 7
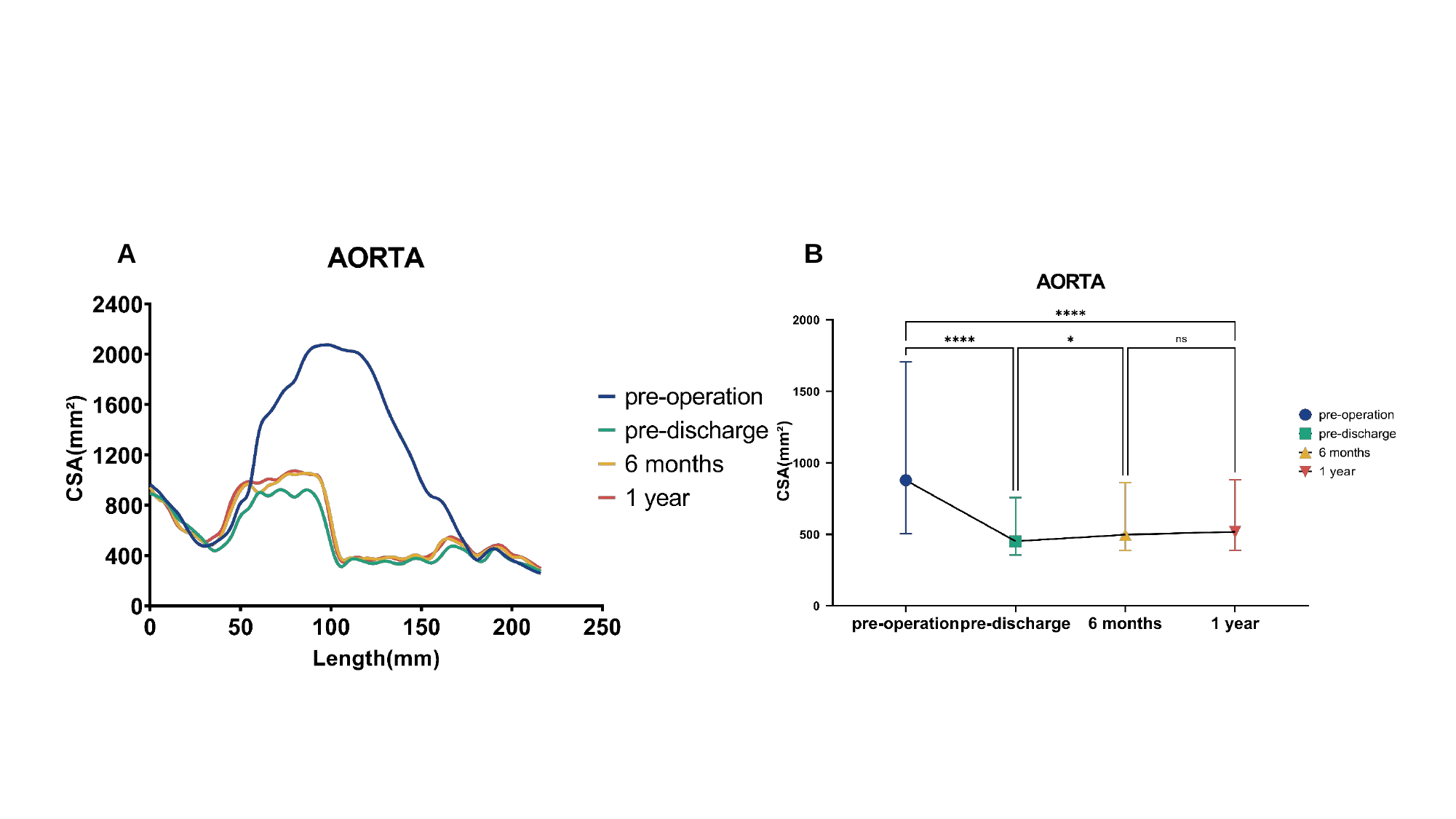

A
B

## Slide 8
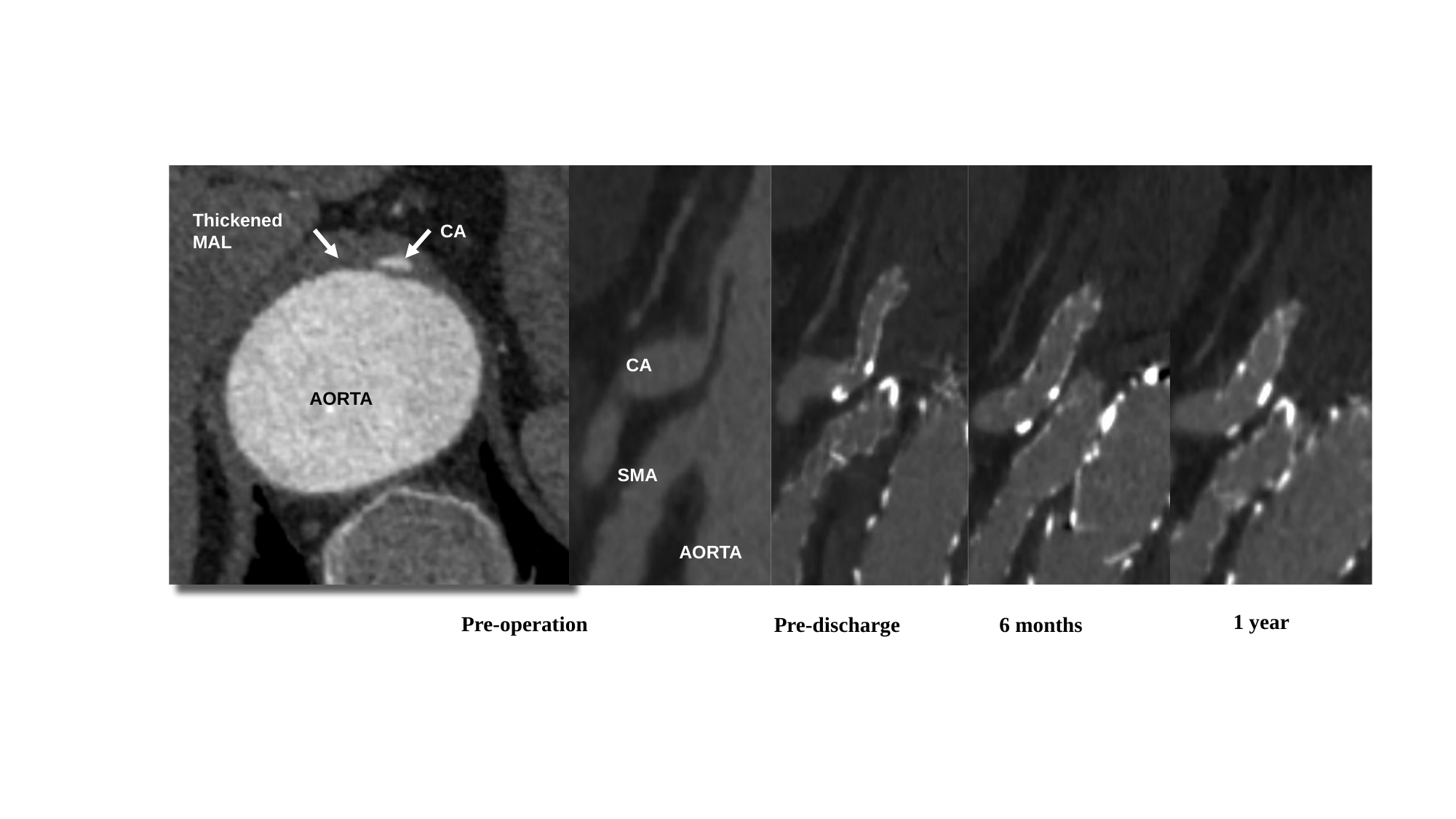

Thickened MAL
CA
CA
AORTA
SMA
AORTA
1 year
Pre-operation
6 months
Pre-discharge

## Slide 9
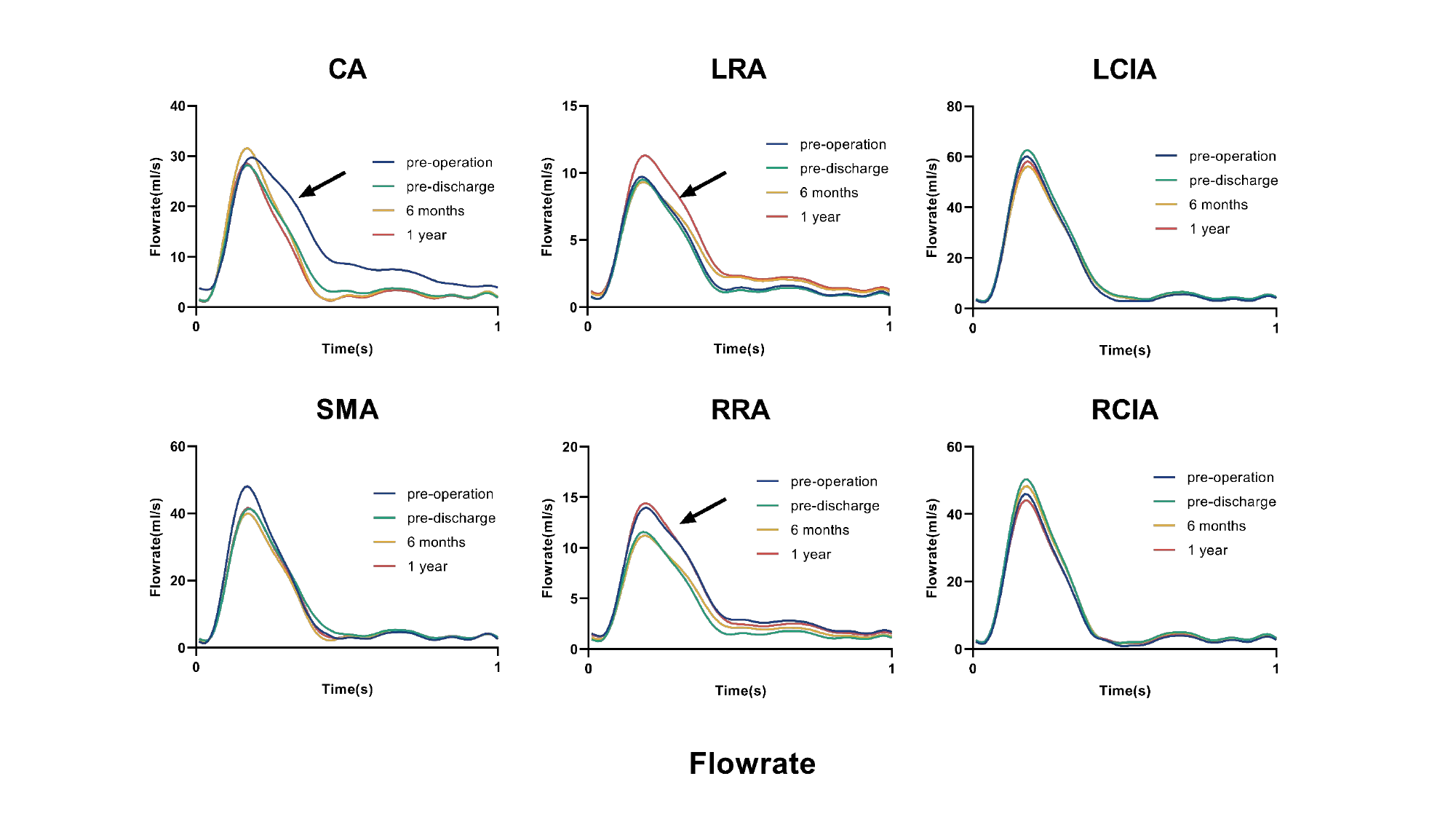

## Slide 10
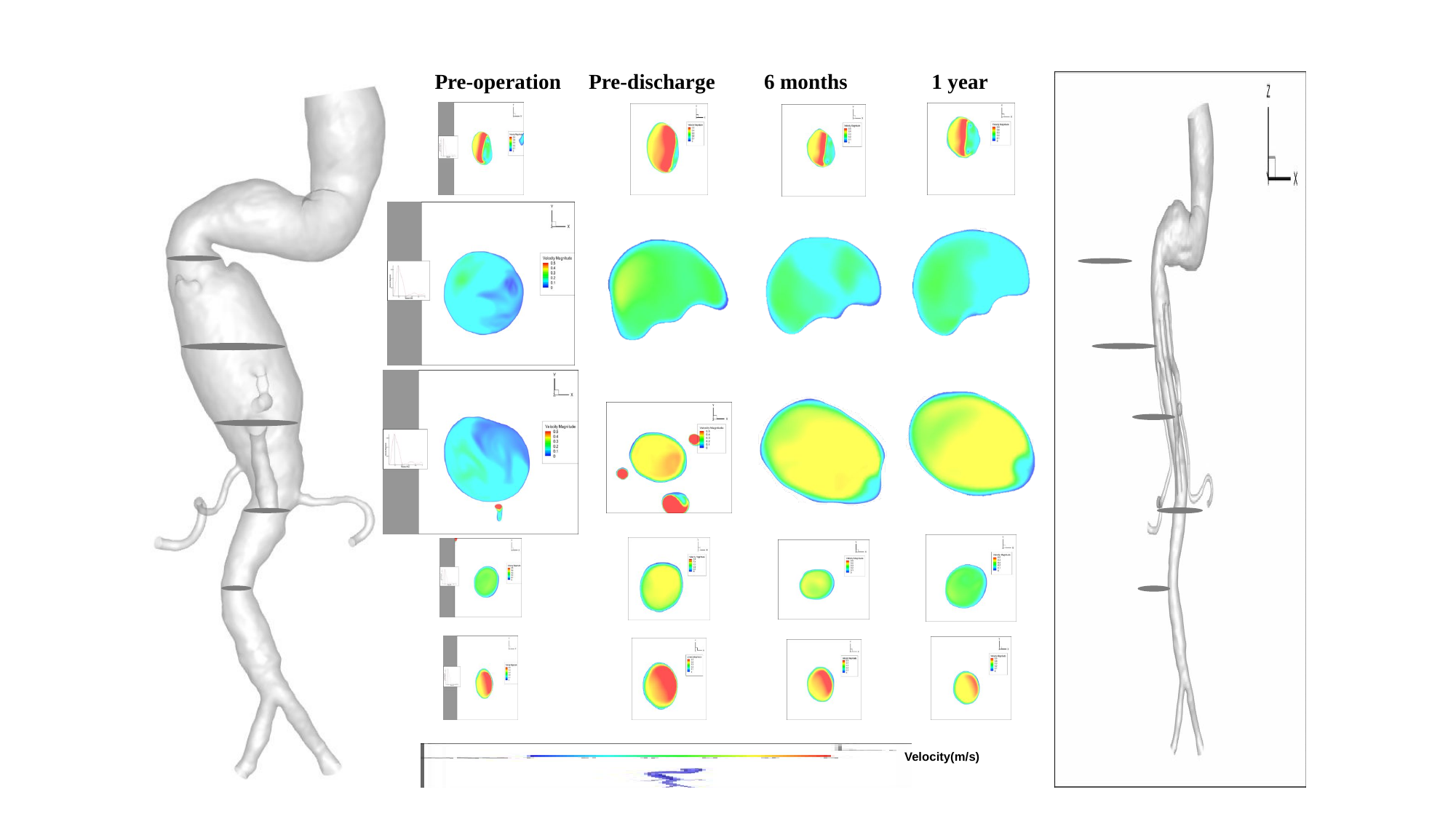

| Pre-operation | Pre-discharge | 6 months | 1 year |
| --- | --- | --- | --- |
Velocity(m/s)

## Slide 11
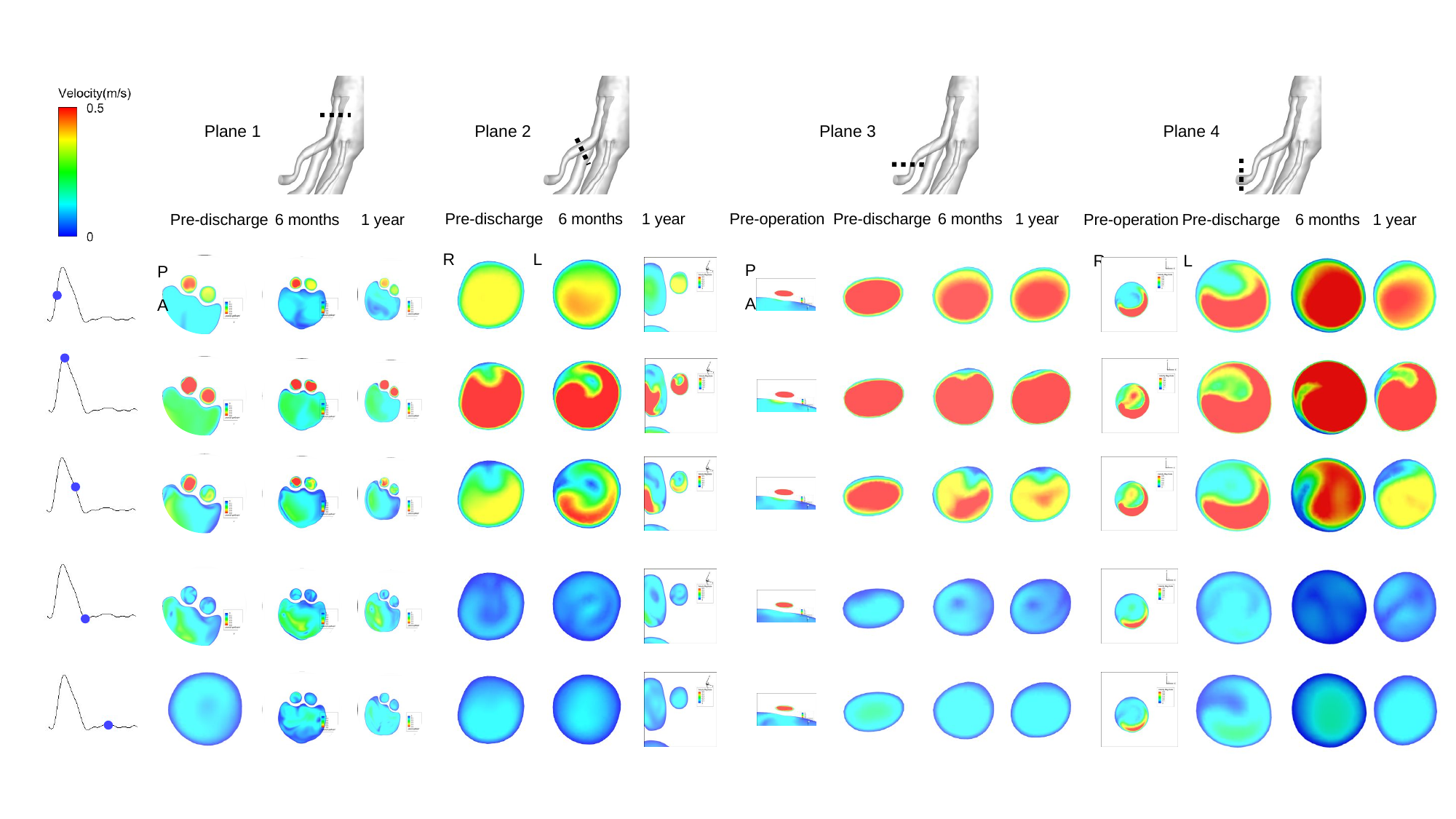

Plane 1
Plane 2
Plane 3
Plane 4
Pre-operation
Pre-discharge
6 months
1 year
Pre-discharge
6 months
1 year
Pre-discharge
6 months
1 year
Pre-discharge
6 months
1 year
Pre-operation
| R | L |
| --- | --- |
| R | L |
| --- | --- |
| P |
| --- |
| A |
| P |
| --- |
| A |

## Slide 12
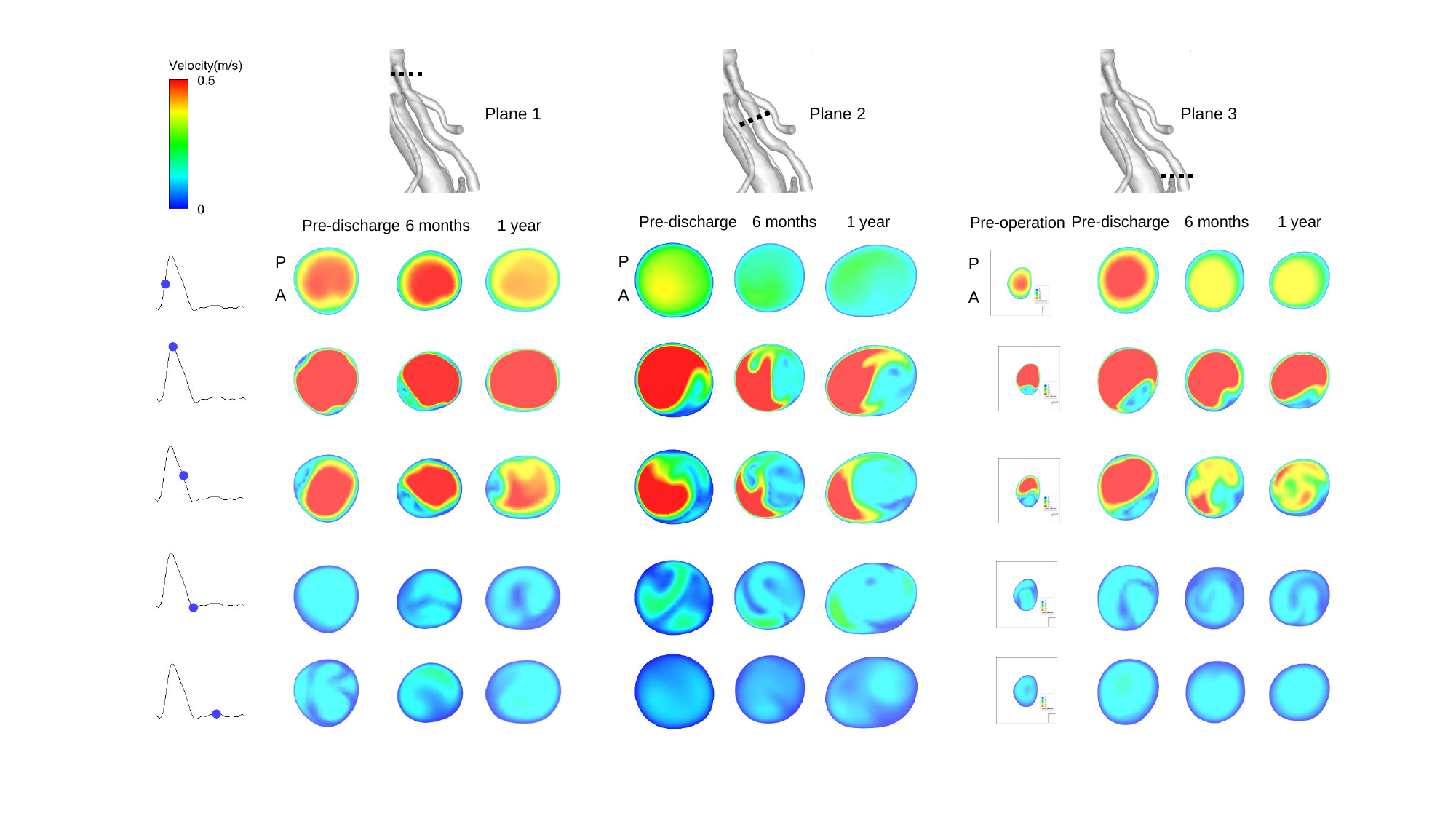

Plane 1
Plane 2
Plane 3
Pre-discharge
6 months
1 year
Pre-discharge
6 months
1 year
Pre-operation
Pre-discharge
6 months
1 year
| P |
| --- |
| A |
| P |
| --- |
| A |
| P |
| --- |
| A |

## Slide 13
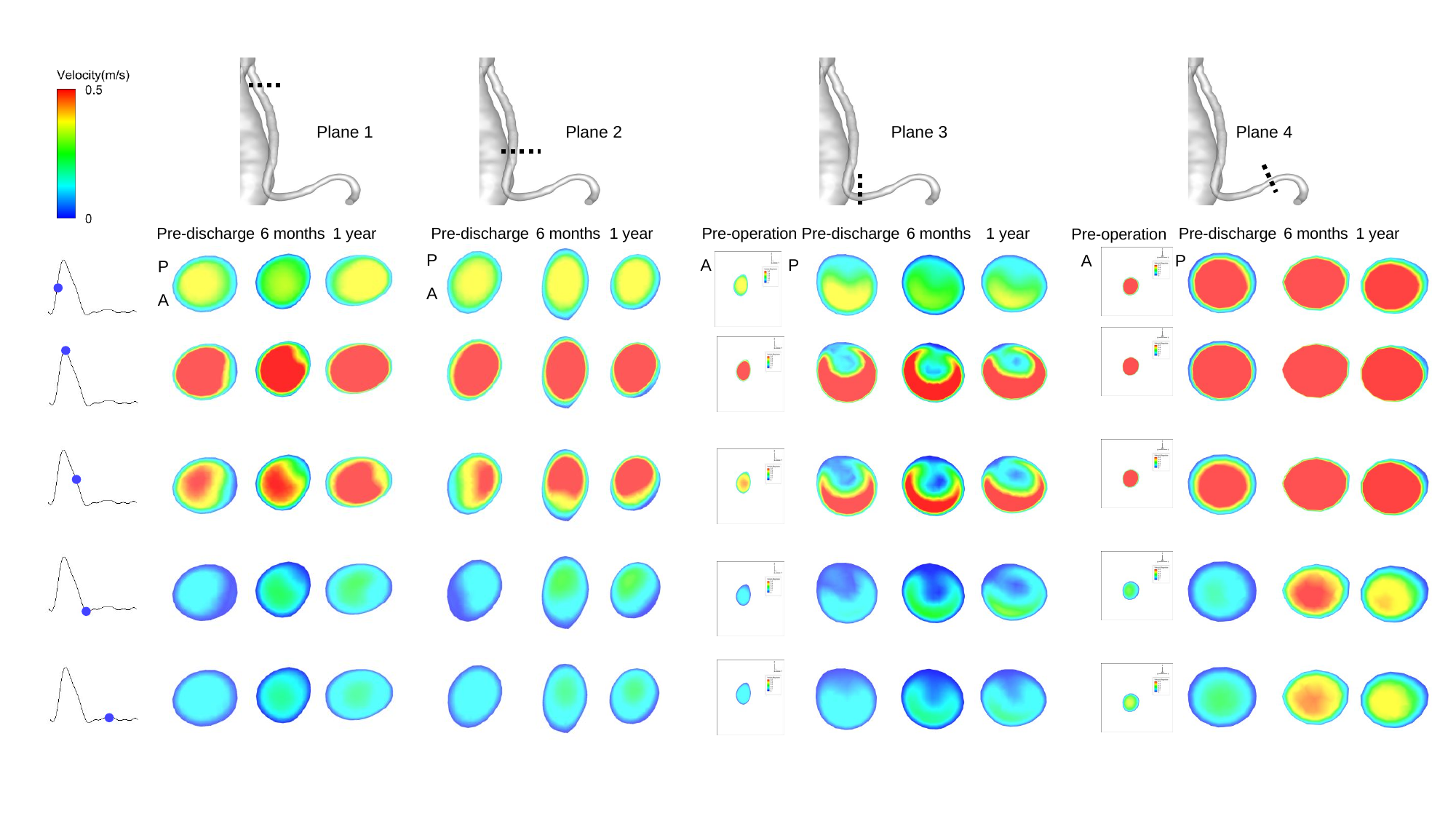

Plane 1
Plane 2
Plane 3
Plane 4
Pre-operation
Pre-discharge
6 months
1 year
Pre-discharge
6 months
1 year
Pre-discharge
6 months
1 year
Pre-discharge
6 months
1 year
Pre-operation
| A | P |
| --- | --- |
| A | P |
| --- | --- |
| P |
| --- |
| A |
| P |
| --- |
| A |

## Slide 14
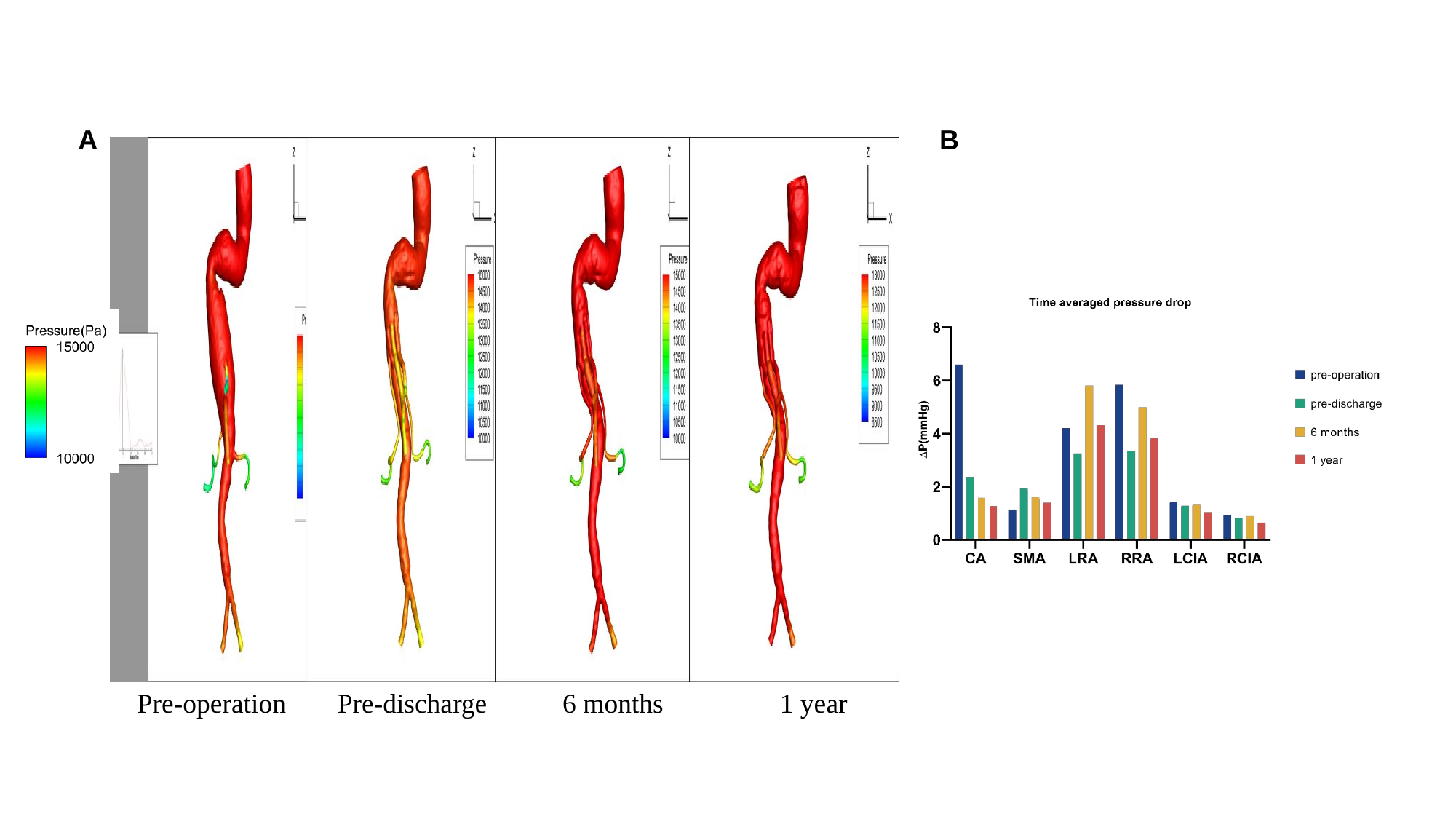

A
B
| Pre-operation | Pre-discharge | 6 months | 1 year |
| --- | --- | --- | --- |

## Slide 15
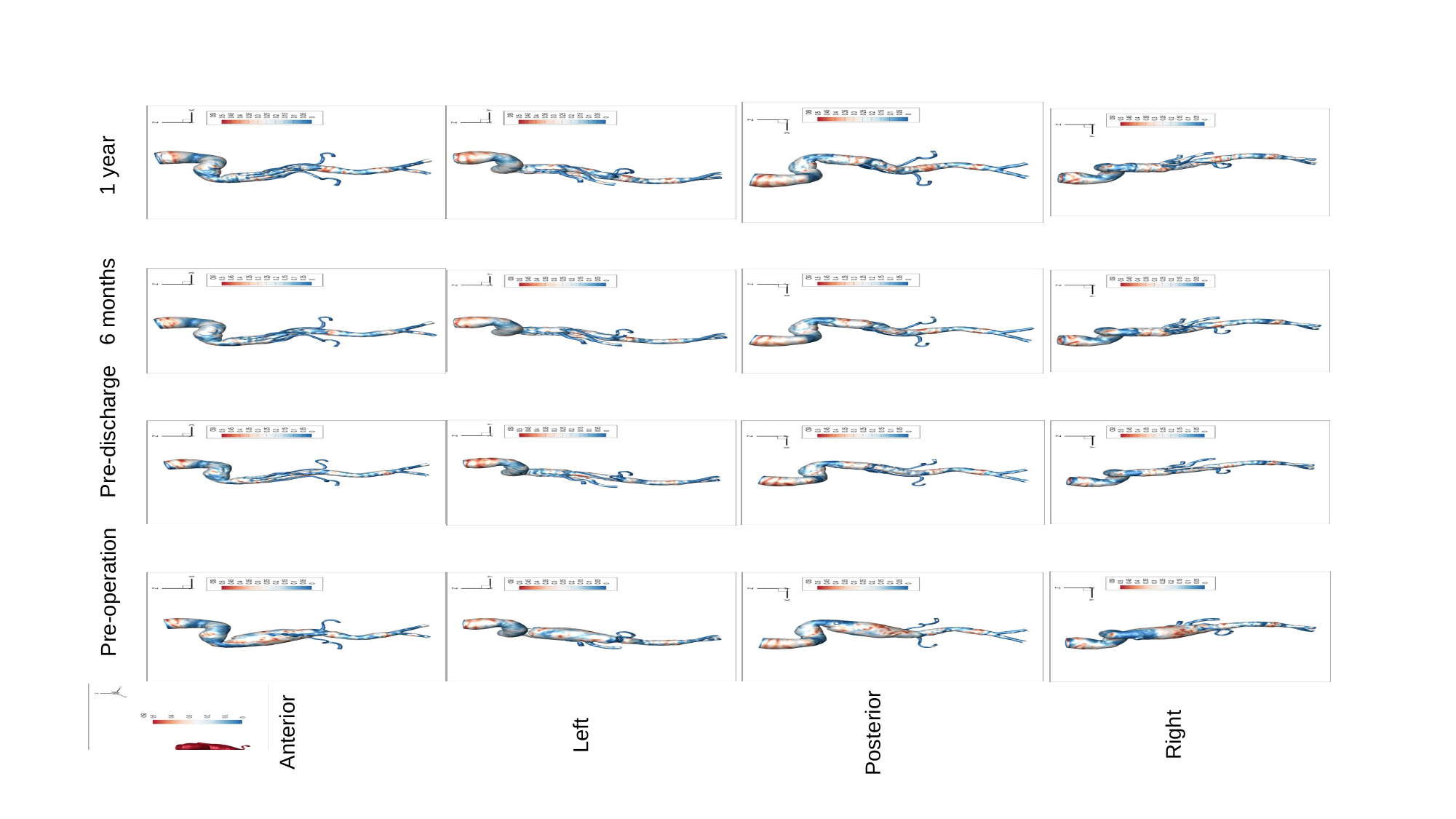

1 year
6 months
Pre-discharge
Pre-operation
Anterior
Left
Posterior
Right
